# Models of Intracellular Transport: Pros and Cons

**DOI:** 10.3389/fcell.2019.00146

**Published:** 2019-08-07

**Authors:** Alexander A. Mironov, Galina V. Beznoussenko

**Affiliations:** Department of Cell Biology, The FIRC Institute of Molecular Oncology, Milan, Italy

**Keywords:** Golgi complex, intracellular transport, COPI, COPII, ER-Golgi transport

## Abstract

Intracellular transport is one of the most confusing issues in the field of cell biology. Many different models and their combinations have been proposed to explain the experimental data on intracellular transport. Here, we analyse the data related to the mechanisms of endoplasmic reticulum-to-Golgi and intra-Golgi transport from the point of view of the main models of intracellular transport; namely: the vesicular model, the diffusion model, the compartment maturation–progression model, and the kiss-and-run model. This review initially describes our current understanding of Golgi function, while highlighting the recent progress that has been made. It then continues to discuss the outstanding questions and potential avenues for future research with regard to the models of these transport steps. To compare the power of these models, we have applied the method proposed by K. Popper; namely, the formulation of prohibitive observations according to, and the consecutive evaluation of, previous data, on the basis on the new models. The levels to which the different models can explain the experimental observations are different, and to date, the most powerful has been the kiss-and-run model, whereas the least powerful has been the diffusion model.

## Introduction

**Figure 1:**
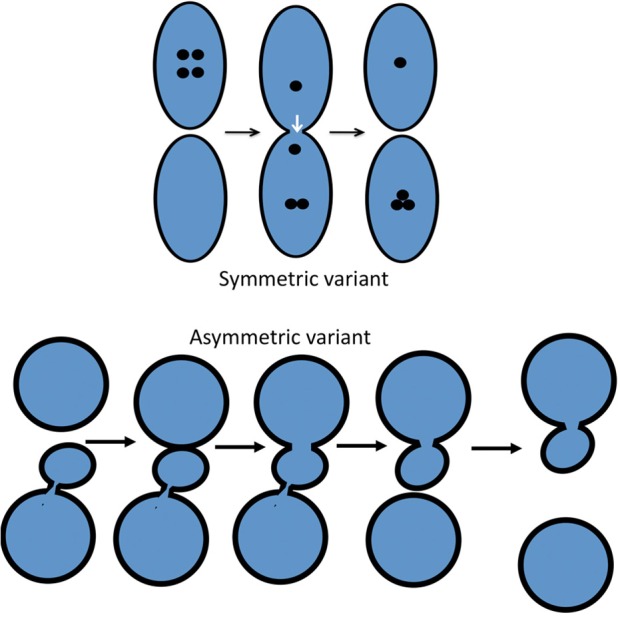
Scheme showing the two variants of the KARM: symmetric and asymmetric. In the asymmetric case (bottom), the cargo domain and a cellular compartment are separated by a thin tubule. Fusion takes place in one site, and fission occurs in another site where this tubule is localized. To function more precisely, it is important to concentrate SNAREs over the cargo domain, which then fuses with the distal compartment (the upper circle).

**Figure 2:**
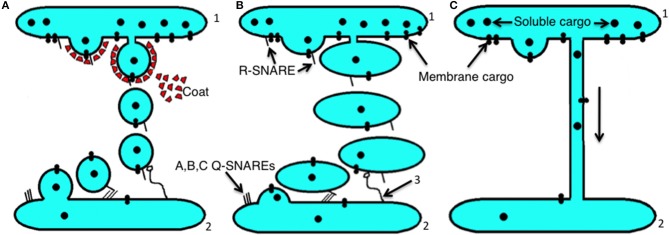
Scheme showing the main principles of intracellular transport. **(A)** The dissociation (mainly vesicular) mode. Initially, membrane buds are formed on a proximal compartment (1) with the help of a protein coat, and then after fission and subsequent uncoating, coat-dependent vesicles move to a distal compartment (2) and are captured by a tethering system (3). Then using SNAREs, the vesicle fuses with the second compartment. **(B)** The progression mode. Initially a large membrane protrusion is formed from a proximal compartment (1), and it undergoes fission. Then this large carrier is captured by the tethering system (3), and with the help of SNAREs it undergoes fusion with a distal compartment (2). **(C)** The lateral diffusion mode of intracellular transport between a proximal compartment (1) and a distal compartment (2).

The structure of the ER–Golgi interface and the Golgi complex (GC) is well-known and has been described many times (Mironov et al., 1998c, 2017; Polishchuk and Mironov, 2004; Mironov and Pavelka, 2008; Klumperman, 2011). Furthermore, most of the molecular machines involved in intracellular transport have now been deciphered (Table 1). Currently, there are four main models of intracellular transport: (1) the vesicular model (VM); (2) the compartment (cisterna) maturation—progression model (CMPM); (3) the diffusion model (DM; Supplementary Figure S1D); and (4) the kiss-and-run model (KARM), which exists as symmetric and asymmetric variants (Figure 1). These models have been well-described in the past (Rabouille et al., 1995; Bannykh and Balch, 1997; Glick et al., 1997; Mironov et al., 1997, 1998a,b; Pelham and Rothman, 2000; Beznoussenko and Mironov, 2002; Luini et al., 2008; Mironov and Beznoussenko, 2008, 2011, 2012; Glick and Nakano, 2009; Pfeffer, 2010; Glick and Luini, 2011; Mironov, 2014). These models are based on three main principles: dissociation, progression, and diffusion (Figure 2; Supplementary Figures S1A–C, S2). Existence of several completely opposite models indicates that there are too many contradictions within this field. In order to solve this problem we used the principle of falsifiability proposed by Popper (1994): any scientific model after its maximal formulation should have a clear description of its so-called prohibitive observations.

**Table 1:** Main molecular machines involved in the ER-to-Golgi and intra-Golgi transport steps (summarized from current reviews).

**Machine**	**Transport step**	
	**ER-to-Golgi**	**Intra-Golgi**
SNAREs (Hong)	Syntaxin5/STX5(Qa-SNARE); GS27/Membrin (Qb-SNARE); BET1 (Qc-SNARE); Sec22(R-SNARE)	Syntaxin5/STX5(Qa-SNARE); GS27/Membrin (*cis-*Qb-SNARE); GS28/GOS28/GOS1R (*trans-*Qb-SNARE); GS15/BETL1 (Qc-SNARE); Yt6(R-SNARE)
Rabs (Lamber et al., 2019)	Rab1a/b; Rab2a/b;	Rab6a/b/c; Rab30; Rab33b; Rab43
COPII (Peotter et al., 2019)	+	–
COPI/ARF (Béthune and Wieland, 2018)	+	+
The multisubunit tethering complexes [TRAPP, Dsl1/Zw10, COG (Smith and Lupashin, 2008; Climer et al., 2018), GARP] (Dubuke and Munson, 2016)	+	+
Cargo receptors (p24 family, ERGIC53, KDELR, TGN46; Stanley, 2011; Cancino et al., 2013)	+	+
Golgins and matrix proteins (Ungermann and Kümmel, 2019)	USO1/p115	USO1/p115; GM130; Giantin; GRASP55; GRASP65; Golgin45; Golgin67; Golgin84; Golgin97/Arl1; Golgin160; Golgin245. GCC185; Syne1; CASP; Bicaudal;…
Glycosylation enzymes and Nucleotide sugar transporters perform glycosylation regulating the transport (Stanley, 2011)		Too many

For the VM, the main falsifying (prohibitive) observation is IGT of megacargoes. For example, the VM poses that IGT is carried out by COPI vesicles. However, COPI vesicles have a diameter of 52 nm, and thus megacargoes cannot be transported by COPI vesicles. However, they are transported (Bonfanti et al., 1998). In order to solve this problem, the megavesicle hypothesis was proposed (Volchuk et al., 2000). In 2001, we showed that megavesicles are not involved in IGT of PCI (Mironov et al., 2001). The second falsifying experiment for the VM of IGT is the depletion of cargo from COPI vesicles.

The prohibitive observations for the DM (see Supplementary Figure S1D) are the following: (1) the concentrating of diffusible cargoes; (2) on each transport step, SNAREs are important; (3) the rarity of connections; (4) the presence of stacks without connections during transport (Trucco et al., 2004); and (5) the deviation from a negative exponential regression line during evacuation of cargo from the Golgi zone. Thus, the DM has significant difficulties to explain the increasing concentrations of cargo proteins (including megacargoes) during EGT and IGT (see below). Also, the DM cannot rationalize the necessity for SNAREs.

Increased concentrations (augmentation of the numeric density) of any cargo, and especially of megacargoes, are the prohibitive observation for the CMPM. Also, the speed of the cargo delivery from *cis-*side to *trans-*side of the GC should be equal. According to the CMPM, all resident Golgi proteins should undergo recycling with the help of COPI vesicles. Thus, depletion of even a few of the resident proteins from COPI vesicles represents the prohibitive observation for the CMPM. The second important restriction of the CMPM is that Golgi-resident proteins should not be depleted in COPI-dependent 52-nm vesicles, because if the concentration of these proteins in the vesicles were lower than in the Golgi cisterna, the recycling of these proteins would be very slow (Glick et al., 1997). Finally, if the Golgi cisternae are immobilized, transport should not be blocked, because of the dynamic nature of Golgi cisternae.

The main principle of the KARM is fusion before fission, and even that fusion results in fission. Fusion/ fission might occur at the same site (i.e., symmetrical variant) or at different sites (i.e., asymmetrical variant). Within the framework of the asymmetrical KARM, fusion would be between the edges of the proximal and distal compartments whereas fission would be somewhere within the proximal compartment where rows of pores or thin tubules should be localized and SNAREs should be concentrated over the cargo domains (Mironov et al., 2013). The KARM does not deny the process of cargo-domain maturation (Mironov et al., 2001). The symmetrical KARM suggests what the concentration mechanism should be; i.e., narrow tubules and asymmetry of ionic composition (Mironov and Beznoussenko, 2012). However, not only narrow, but also relatively thick connections can induce increased cargo concentrations in one of two compartments. If the delivery of protons is asymmetric because the diffusion of protein aggregates backwards is slower than in the anterograde direction. Fusion and fission should have molecular mechanisms for their realization (Supplementary Figure S2).

The prohibitive observations for the KARM are the following:

1. Membrane cargoes and megacargoes should be organized in domains. Large cargo domains are more effective for transport than vesicles. It is necessary to have the concentrating of SNAREs over cargo domains.
2. SNARE should be concentrated over cargo domains. The concentrating of cargoes and SNAREs increases efficiency. Pores or thin tubules behind a cargo domain provide directionality.
3. Between the cargo domain and the proximal compartment there should be a thin membrane tubule(s), which connects a cargo domain with the compartment domains *per se*. Also, this demand might be fulfilled by a row of pores. For fission, it is necessary to have the fission machinery, like BARS (Yang et al., 2005), endophilin (Yang et al., 2005), ARFGAP (Yang et al., 2002), and different organization of the lipids (i.e., the membrane of COPI buds is thinner than in a cisterna; Orci et al., 1996).
4. Pores should be consumed during transport.
5. There should be a negatively exponential regression line for the process of emptying of the GC *per se* under all conditions.
6. For EGT and post-Golgi transport, the bolus-like mechanism should be optimal.
7. All of the compartments should always be connected. However, the existence of many SNAREs already denies this falsification observation.
8. When secretory compartments cannot attach to each other. Previously the KARM could not explain IGT in *S. cerevisiae*, where all of the Golgi compartments are localized far away from each other. However, we then described connections between different Golgi compartments (Beznoussenko et al., 2016), and next, Kurokawa et al. (2019) reported that the cargo and the Golgi domains can be situated within the same perforated membrane disk.

## ER-Golgi Transport

### The Vesicular Model

**Figure 3:**
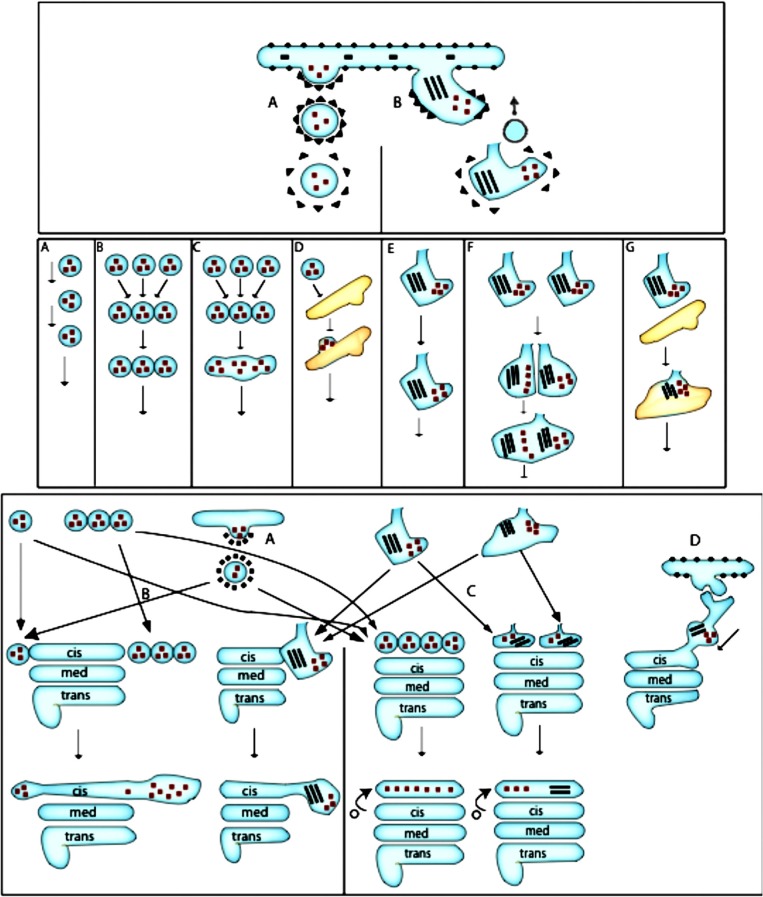
Scheme of ER-to-Golgi transport according to the VM and CMPM. Upper section: **(A)** Formation of COPII-coated bud. **(B)** Formation of a COPII-dependent protrusion, which might be partially coated with COPII, for a megacargo inside this protrusion. A COPI vesicle recycles the resident ER proteins. Middle section: **(A)** Movement of COPII-dependent vesicles one after another toward the Golgi complex. **(B)** Formation of a vesicle aggregate that moves toward the Golgi complex. Fusion of a COPI vesicle with the formation of a ER-to-Golgi carrier. **(D)** Delivery and fusion of the COPII vesicle to the intermediate compartment. **(E)** Movement of the ER protrusion. **(F)** Fusion of the ER protrusion, with the formation of common carrier. **(G)** Fusion of the protrusion-dependent ER-to-Golgi carrier with the intermediate ERGIC compartment. Lower section: Different possibilities for the final delivery of ER-to-Golgi carriers to the Golgi complex. Left: According to the pure VM: **(A)** The additional round of COPII-dependent vesicle formation from the ER-to-Golgi carrier from the intermediate compartment. **(B)** Delivery of a new vesicle to the Golgi complex. **(C)** According to the CMPM: COPII vesicles or ER protrusions form the new *cis-*Golgi cisterna. **(D)** According to the bolus-like mechanism within the framework of the KARM: Movement of the carrier along the ER-Golgi tubule.

The VM of EGT poses that the exit of cargo proteins occurs in COPII-coated buds. These buds undergo fission and form COPII-coated spherical vesicles (Antonny et al., 2003; Figure 3A in Lee et al., 2005; Brandizzi and Barlowe, 2013; Zanetti et al., 2013; Saito and Katada, 2015). After separation of their coating, these vesicles are transported to the GC as individual vesicles or vesicle aggregates (Bannykh et al., 1996). There are several variants of the VM of EGT (Figure 3). Cargo receptors such as TANGO1 are important for some cargoes.

The main support for the VM of EGT was the study by Kaiser and Schekman (1990). However, their interpretation already contained contradiction, because during intracellular transport, as well as COPII vesicles being generated, COPI vesicles should also be generated (according to the VM of IGT, these execute IGT). So COPI vesicles should appear, and their numbers should be higher than that COPII vesicles because their volume is smaller. We found another explanation of their results (see Supplementary Materials).

The second corner-stone study assumed to be in favor of the VM is that of Barlowe et al. (1994). They isolated COPII-coated vesicles after incubation of yeast microsomes with purified component of COPII in the presence of GTP. Importantly, after incubation of microsomes with COPII subunits and GTP, they obtained a mixture of tubules and vesicles. They then filtered this membrane fraction through a gel with small pores. They concluded that these vesicles are formed by COPII and that these vesicles contain cargo proteins. Finally, Bednarek et al. (1995) described so-called COPII-coated buds on the ER of *S. cerevisiae* after cell permeabilization and incubation with COPII subunits.

There is no doubt that COPII is important for the exit of several cargoes from the ER (Aridor and Balch, 2000; Lee and Linstedt, 2000; Mironov et al., 2003; Omari et al., 2018). Cargo moving from the rough ER to the GC passes through several stages: COPII-, COPII/COPI-, and COPI-co-localization, to finally undergo centralization (Scales et al., 1997). COPII concentrated cargo localized within the artificial membrane (Tabata et al., 2009). Recently, Kurokawa and Nakano (2019) showed that ERES are specialized ER zones for transport of cargo proteins from the ER to the GC. Of interest, in our chapter in a Golgi book (see Figure 1; page 18 of Mironov and Pavelka, 2008), round profiles could be seen localized within ERES, with indications that this profile represents COPII vesicles. Thus, previously we believed that COPII vesicles exist.

In spite of the importance of COPII, EGT occurs even in the absence of COPII (reviewed by Mironov, 2014). For example, Bard et al. (2006) showed that EGT still occurs after elimination of COPII subunits, although at a slightly lower rate (54% of the control). Insect cells lacking Sec13 can divide at a normal rate (Tsarouhas et al., 2007). Caco-2 cells lacking Sec13 (in humans, this protein has only one isoform) can grow (Townley et al., 2012). That these cells can divide suggests that the delivery of membrane to the plasma membrane necessary for membrane duplication during cell division was normal. Thus, in the absence of Sec13, Caco-2 cells can transport membrane proteins. There is a lack of detectable defects in the transport or secretion of small, soluble, freely diffusible proteins or transmembrane proteins in Sec13-suppressed cells (Townley et al., 2008) and Caco-2 cells (Townley et al., 2012). Deletion of COPII subunits (i.e., Sar1A, B isoforms, or both isoforms of Sar1p, together with both isoforms of Sec23) did not inhibit EGT of soluble and membrane cargoes (Cutrona et al., 2013). Elrod-Erickson and Kaiser (1996) demonstrated that in *S. cerevisiae*, deletion of Sec13 together with one of the proteins “bypass of Sec13” (BST)1, BST2 (also known as EMP24, the p24 family protein) or BST3 did not cause cell death, although the removal of just Sec13 was lethal for the cells. In the absence of Sec23 and also of p24, which regulates the behavior of COPI, transport occurs in yeast. Also, in the absence of cargo transport, the GC in yeast disappears (Ayscough and Warren, 1994; Morin-Ganet et al., 2000). The VM, CMPM, and DM cannot explain this phenomenon. In contrast, the KARM can (Mironov et al., 2013). Moreover, to date, nobody has demonstrated that under normal conditions, such as at steady-state, membrane buds on the granular ER and presumably coated, contain Sec13 in their coats. Also, nobody has showed how COPII vesicles or aggregates of COPII vesicles (as was proposed by Bannykh et al., 1996) move toward the GC. We did not find membrane buds on the ER in *S. cerevisiae* (Beznoussenko et al., 2016). Other studies have also not demonstrated the presence of these buds (reviewed by Beznoussenko et al., 2016). Importantly, according to the most precise electron cryo-microscopy (Bacia et al., 2011), the diameter of COPII vesicles is 65–80 nm. Importantly, COPI vesicles in *S. cerevisiae* have a diameter of 50 nm (Beznoussenko et al., 2016). The vesicles accumulated by Kaiser and Schekman (1990) also had a diameter of 50 nm.

However, the main contradiction to the VM is the exit of megacargoes, such as pre-chylomicrons [in enterocytes (Sabesin and Frase, 1977; Siddiqi et al., 2003)], very low-density lipoprotein (VLDL) [in hepatocytes (Claude, 1970)] and procollagen aggregates (Figures 9–11 in Karim et al., 1979; Bonfanti et al., 1998; Mironov et al., 2003; Patterson et al., 2008). The list of megacargoes includes not only PCI (Supplementary Figure S1, on the right), chylomicrons and VLDL, but also relatively large viruses, virions of which are formed within the nuclear envelope or inside the ER and have diameter of up to 200 nm. For instance, *Herpes* virus is formed within the nuclear envelope and then is transported toward the GC. Using high-resolution electron microscopy, COPII coated buds were not seen on the granular ER, with no COPII-coat in the ER domain where virions were localized (Wild et al., 2017). Also, separated vacuoles with a virion inside their lumen were not detected. They suggested that transport is realized according to a bolus-like mechanism along the ER tubule (see Figure 11 of Wild et al., 2017). It was shown that aggregates of PC are formed already inside the lumen of the ER cisternae (Figures 9–11 of Karim et al., 1979; see also Mironov et al., 2003). Chylomicrons and VLDLs are also formed inside the lumen of the ER (Claude, 1970; Sabesin and Frase, 1977). Several virions are formed inside the lumen of the nuclear envelope.

Megacargoes cannot be inserted into 65–80 nm COPII-dependent vesicles. It is also highly unlikely that megacargoes disassemble into smaller subunits that can be packaged into conventional transport vesicles. For instance, the HSP47 protein helps PCI to form rigid 300-nm trimers already in the ER, and the environment in the GC is not suitable for their disassembly (Bruckner and Eikenberry, 1984; Bonfanti et al., 1998; Patterson et al., 2008). Similarly, chylomicrons and VLDL are formed inside the smooth ER and cannot be fragmented in the GC. Moreover, at the electron microscopy level, there is no coat visible on the distensions of the ER that contain megacargoes (Mironov et al., 2003). Of interest, Sec13 depletion impaired the deposition of large ECM components such as collagen (Townley et al., 2008). In the absence of COPII, exit of procollagen from the ER is inhibited (Cutrona et al., 2013). Furthermore, not only the absence of a COPII coat, but also the slowdown (after depletion of Sedlin; Venditti et al., 2012) or acceleration of COPII turnover (the Sec23A-M702V mutation, Kim et al., 2010) inhibited procollagen exit from the ER.

To solve these contradictions, it has been proposed that large cargoes are transported by “megavesicles,” or “megacarriers” that are formed by unusual combinations of isoforms of COPII subunits (Fromme and Schekman, 2005; Venditti et al., 2012; Malhotra et al., 2015; Santos et al., 2016; Gorur et al., 2017; Raote et al., 2017, 2018). It was proposed that mono-ubiquination of Sec31 can enlarge the COPII coat to accommodate collagen fibrils (4 × 300 nm; Jin et al., 2012). According to the megavesicles model, large cargo aggregates form in megabuds coated with COPII. It has been shown that the membrane protein TANGO1 binds PCVII, and that TANGO1 binds the COPII coat proteins Sec23/Sec24 (Saito et al., 2009). Knockdown of TANGO1 inhibits export of the bulky PCVII (but not of PCI) from the ER (Saito et al., 2009; Nogueira et al., 2014). However, to demonstrate that megabuds and megavesicles exist, it is necessary to show buds coated with COPII or separated megavesicles coated with COPII (preferably using correlative light-electron microscopy or immune electron microscopy). If megavesicles exist, Sec13 should form a cap over the procollagen aggregate, VLDL or chylomicron. Of interest, to date, this requirement has not been fulfilled. For instance, Santos et al. (2016) did not demonstrate co-localization between Sec13 and lipids or Apo B (lipid and Apo proteins of VLDL). There is not any coat visible at on the distensions of the ER filled with procollagen (Mironov et al., 2003). Claude (1970) and Sabesin and Frase (1977) have not observed pre-chylomicrons in the ER megabuds. Of interest, in Figure S1f (by Subramanian et al., 2019), PCI aggregates are larger than TANGO1- positive spots. TANGO1-positive spots do not form rings around PC aggregates and not significant overlapping of TANGO1 and PC was observed. This suggests that TANGO1 could function as the center of PC aggregation (crystallization) and not as a mechanism involved into the formation of COPII coat. Importantly, Santos et al. (2016) did not discuss the contradiction of their data by the results of Siddiqi et al. (2003). It was shown that aggregates of procollagen are formed inside the lumen of the ER cisternae (Figures 9–11 of Karim et al., 1979). However, no coat was visible over these distensions of the ER. Raote et al. (2018) do not provide any single image that directly confirms the scheme of the formation of megabuds proposed by the authors, namely, the COPII ring, then the more external ring of TANGO1, and procollagen-positive spots inside these rings. Thus, at steady-state, megabuds that contain procollagen and are coated with a COPII-like coat were also not detected (Leblond, 1989).

To demonstrate that megavesicles exist, Gorur et al. (2017) engineered cells to stably overexpress the human pro-α1 (I) collagen. Using correlative light-electron microscopy based on serial sections with a thickness of 70–100 nm, they demonstrated a structure filled with PCI with a diameter of 900 nm (Figure 2C: Z7 of Gorur et al., 2017). The thickness of the coat over this structure was more than 40 nm, whereas the typical thickness of COPII coats is 12 nm (Bannykh et al., 1996; Bacia et al., 2011). Also, the significant thickness of the serial sections indicates that the resolution along the Z-axis was 140–200 nm. Therefore, it is not possible to judge whether this structure was connected with the ER or not. Moreover, in the vast majority of studies, the diameter of the procollagen-containing ER-to-Golgi carriers or Golgi cisterna distensions filled with procollagen never exceeds 350 nm (Leblond, 1989; Bonfanti et al., 1998; Patterson et al., 2008; Perinetti et al., 2009). Importantly, in Figure 2ii of Gorur et al. (2017), the labeling for Sec31a does not form a ring, as it should do to be in agreement with the hypothesis of COPII-coated megavesicles. Of interest, the area of the labeling for procollagen is wider than the labeling for Sec31A; namely, near the border the green intensity is higher than the red intensity, whereas in the center the intensities of red and green are equal. Gorur et al. (2017) used super-resolution light microscopy to detect the ring- or cap-like labeling for Sec31A, which should surround the collagen aggregate. Moreover, in their Figures 3A v–x, the thickness of the COPII coat is >100 nm, whereas under normal conditions, the thickness of the COPII coat is only 12 nm (Bannykh et al., 1996). Moreover, the diameter of the procollagen aggregate was only 100 nm, although under normal conditions their diameter is 300 nm (Mironov et al., 2003). Also, there is an empty space between the PCI spot and the Sec31A-positive cap (see Figure 3A viii of Gorur et al., 2017). Such a space has never been observed under normal conditions. On the other hand, in their Figures S5B:i–iii (Gorur et al., 2017), the diameter of the vesicles is about 200 nm. Only in Figure S5B:iv do the vesicle have a diameter of 350 nm, although this vesicle was not coated. Similar large procollagen-positive immobile dots were observed by Omari et al. (2018). Also, McCaughey et al. (2019) observed huge procollgen-positive spots (their Figure 2), which did not go to the GC.

Recently, McCaughey et al. (2019) provided direct evidence that they suggested was in favor of the very minor (if any) role of megavesicles for EGT of procollagen. They demonstrated that EGT of procollagen occurs without the formation of COPII-coated 200–300 nm carriers. These observations contradict the megavesicle hypothesis proposed by the proponents of the VM of EGT. However, these authors did not discuss this issue, and simply claimed that their “data are consistent with COP II-dependent trafficking of procollagen” (McCaughey et al., 2019, page 12). Also, they did not cite two important papers by Patterson et al. (2008) and by Mironov et al. (2003), where the mechanisms of EGT of PCI were described and the role of COPII was questioned. Moreover, Patterson et al. (2008) presented data on PCI transport in live cells, whereas we had already demonstrated the rarity of the arrival of ER-to-Golgi carriers with a diameter of 300 nm at the GC. Finally, in contrast to McCaughey et al. (2019), we observed rare ER-to-Golgi carriers filled with procollagen III that arrived at the Golgi area after GFP bleaching (Beznoussenko et al., 2014). In McCaughey et al. (2019), the procollagen-containing dots grew inside the Golgi area. In our studies, these dots have acquired their high brightness at the periphery, and then moved to the Golgi area.

Also, Patterson et al. (2008) demonstrated ER-to-Golgi carriers containing procollagen and moving toward the GC. The size here was <350 nm. Analysis of Movie S1 by Omari et al. (2018) revealed that after bleaching of the ER, which was filled with procollagen, within the area near the GC the spots containing concentrated procollagen moved toward the GC, which was labeled with GM130. Omari et al. (2018) claimed that they observed procollagen-positive spots initially coated with COPII, which after uncoating, moved toward the GC However, careful analysis of Movie S2 revealed that the procollagen-positive spot is formed not within the domains that co-localize with Sec23, but near the Sec23-positive blob at a distance of about 200 nm. In Movie S2, the Sec23-positive rather big spot shown with a back and forth movement, when suddenly a spot containing concentrated labeling for procollagen appeared near the Sec23-positive spot. This uncoated spot then starts to move toward the GC. Sec23-positive rings that surround procollagen-positive spots were not shown. This event occurred exactly as it was described in our study (Mironov et al., 2003). There, we showed that procollagen spots are formed not within ERES, but nearby. No image is shown that demonstrates procollagen-positive spots surrounded with Sec23-positive ring, as derived from the VM of EGT. Significantly, the overall numbers decreased for both LC3-positive autophagic structures and FP-LC3–positive autophagic structures that contained FP-proα2G610C(I) (Omari et al., 2018).

### The Diffusion Model

Within the framework of the DM, EGT occurs by diffusion along constant connections between the ER and the GC. The precise characteristics of the DM of EGT have not yet been specified in the literature. Direct membrane continuities between the ER and the GC have been described many times (Flickinger, 1969, 1973; Claude, 1970; Maul and Brinkley, 1970; Bracker et al., 1971; Holzman, 1971; Morre et al., 1974; Franke and Kartenbeck, 1976; Novikoff and Yam, 1978; Uchiyama, 1982; Broadwell and Cataldo, 1983; Sasaki et al., 1984; Lindsey and Ellisman, 1985; Williams and Lafontane, 1985; Lockhausen et al., 1990; Krijnse-Locker et al., 1994; Sesso et al., 1994; Mironov et al., 2017; also reviewed in Mironov and Pavelka, 2008). These observations were based on high voltage electron microscopy (Lindsey and Ellisman, 1985), scanning electron microscopy (Tanaka et al., 1986), three-dimensional reconstruction of serial sections (Sesso et al., 1994), functional analysis of transport (Krijnse-Locker et al., 1994), and electron microscopy tomography (Ladinsky et al., 1999). For instance, Ladinsky et al. (1999) described a connection between the ER and a small cisterna that showed all of the features of Golgi cisternae; namely, buds and small pores (see Figure 3: C6′ of Ladinsky et al., 1999). They interpreted this structure as “the specialized domain of the ER.” However, such small pores were never observed in the ER cisternae (see Figure 3: *cis-*ER,:*trans-*ER of Ladinsky et al., 1999). Moreover, to date, nobody has confirmed the possibility that the ER cisterna can be inserted into a Golgi stack. Fixative cannot generate membrane tubules. Moreover, the fixative usually disrupts pre-existing tubules (Duman et al., 2002). Importantly, connections between the ER and pre-Golgi carriers have been described even after application of quick-freezing (Mironov et al., 2003). Previously, we also reported such connections after depletion of both isoforms of Sar1 (Cutrona et al., 2013).

Trucco et al. (2004) (see their Figures 1d,e) demonstrated connectivity between the ER and the *cis-*most cisterna (CMC) after a 15°C temperature block. However, the existence of ER–Golgi connections was not confirmed by Koga and Ushiki (2006). Finally, at 15°C, ERGIC53/58, the KDEL receptor, SNAREs operating at the level of the intermediate compartment, members of the p24 family, and even Man I, redistribute from the pericentral GC to the peripheral spots labeled for COPI/COPII (see above). However, at this temperature, vesicular transport is blocked (Saraste and Kuismanen, 1984; Kuismanen and Saraste, 1989), which suggests that these molecules diffuse along the membrane continuity from the pericentral Golgi to ERES.

### The Cisternal Maturation–Progression Model

According to the CMPM, immature ER-to-Golgi carriers are formed by protrusion from the ER, whereas ER-resident proteins are eliminated from the ER-to-Golgi carriers by retrograde COPI-dependent vesicles (Mironov et al., 2003; see their Figures 2, 3B, upper part). The main argument in favor of the CMPM of EGT is the data of Oprins et al. (2001). They observed a significant (57.6-fold) higher aggregation of chymotrypsinogen and proposed “the concentration by exclusion” (Supplementary Figure S1, lower part). However, COPI vesicles that presumably operate as carriers for retrograde transport at the level of ERES cannot be used because COPI vesicle have a very high ratio between their surface and volume, which means that these vesicles are not suitable to explain such high levels of cargo concentration. Recycling of COPI vesicles would eliminate mostly the surface area but not the volume of the immature ER-to-Golgi carriers. Moreover, the simultaneous increase in the amylase aggregation was only 3.7-fold suggesting that several types of COPI vesicles (for each cargo) operate at the level of ERES. However, this has not yet been demonstrated. On the other hand, spots carrying fluorescence cargoes toward the GC do not increase the intensity of their fluorescence during the centripetal movement, and no small spots have been seen to detach from them during this movement (Presley et al., 1997; Scales et al., 1997; Stephens et al., 2000). These suggest that the elimination of membranes not containing cargo proteins does not occur.

If COPI-coated vesicles mediate retrograde, Golgi-to-ER, transport, the concentrating of proteins with KKXX motifs would be expected, such as ERGIC53/58 or p24, in COPI-coated buds. However, to date, there has been no convincing evidence that demonstrates the concentrating of either ERGIC53/58 or p24 in COPI-coated buds on ERES. Moreover, alpha 2 protein of the p24 family is not enriched in Golgi buds (Dominguez et al., 1998). ERGIC53 (Gilchrist et al., 2006) and Hsp47 are depleted in COPI-dependent vesicles that are formed also within ERES (Bannykh et al., 1996). ERGIC53/58 and proteins of p24 family are not concentrated in small, coated round profiles observed near the GC (Cole et al., 1996; Jäntti et al., 1997) or isolated in the presence of GTP (Stamnes et al., 1995, 1998; Rojo et al., 1997, 2000; Bremser et al., 1999). In the single report where ERGIC53 was found in round profiles in the Golgi area (Palokangas et al., 1998), they did not present serial sections. The microinjection of the Sar1p:GTP-restricted mutant induces redistribution of ERGIC proteins to the ER. However, after the microinjection of Sar1p:GTP-restricted mutant together with the inhibitory antibody against ßCOP, the ERGIC53/58, KDEL receptor, cholera toxin, and p24 proteins did not redistribute to the ER, whereas Shiga toxin was shifted to the ER. To explain these observations, two pathways for Golgi-to-ER transport were proposed; namely, COPI-dependent and COPI-independent (Girod et al., 1999). We think that careful analysis of the concentrating of the resident ER and ERES proteins in COPI vesicles derived from ER-to-Golgi carriers might solve this prohibitive observation (this concentration should be higher than in the ER-to-Golgi carriers) or confirm the CMPM of EGT.

### The Kiss-and-Run Model

**Figure 4:**
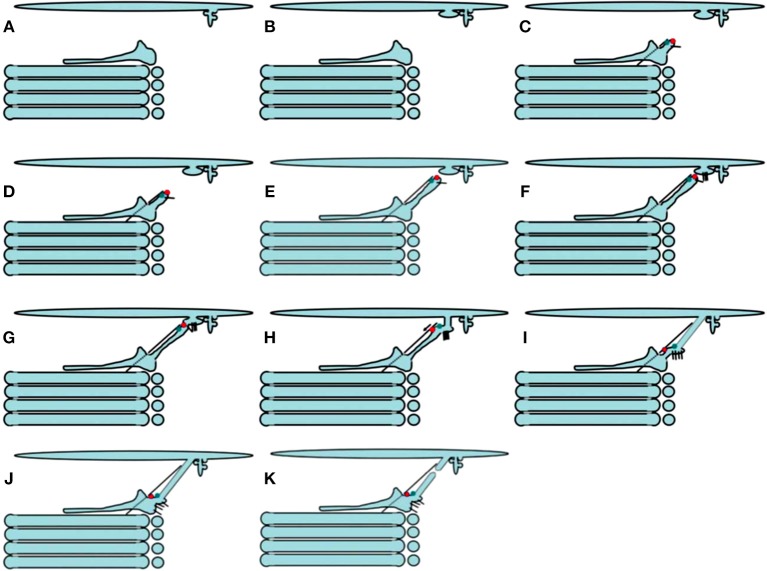
Scheme of ER-to-Golgi transport according to the KARM. Protrusions containing small cargoes **(A)** and megacargoes **(B)** are formed within the ERES area. **(C–F)** A tubule is formed from the *cis-*most Golgi cisterna. It moves toward the ERES area along a microtubule (dashed line), with the help of kinesin (green dot). **(G,H)** This tubule fuses with ERES with the help of SNAREs (short black lines), and delivers dynein (red dots) to the ER-to-Golgi carrier. **(I,J)** The bolus moves toward the Golgi complex using dynein and microtubules. **(K)** Now, the ER-to-Golgi carrier is within the *cis-*most cisterna. Finally, the tubular connection undergoes rupture **(K)**.

The characteristics of the KARM of EGT have not yet been specified in the literature. Here, we proposed our variant of the KARM of EGT (Figure 4). The KARM assumes that EGT is realized by a fusion-fission mechanism: initially the membrane protrusion filled with a cargo is formed, and then this protrusion fuses with a tubule that emanates from the GC, and in particular from the CMC. If the distance between the proximal and distal compartments is large, the distal compartments are extended to the proximal, and capture the cargo domain. This was shown by Casler et al. (2019). After this fusion, the fission occurs near the neck that connects the protrusion and the ER. The tubule delivers dynein to the immature ER-to-Golgi carriers. This motor moves the ER-to-Golgi carriers toward the GC. The arrival of this carrier at the GC generates flux of Ca^2+^ from ERES (Micaroni et al., 2010a,b) and stimulates fusion of the carrier with the medial GC. When two consecutive compartments are localized far away from each other, the KARM suggests the need for a bolus-like mode of transport. The bolus model was originally proposed for exocytosis (Ayala, 1994). It implies participation of active peristaltic movement of membrane varicosities using mechanical forces generated by membrane coats located at the proximal side of the bolus. The main postulate of the asymmetric variant of the KARM, which is the only one useful for membrane transport (for soluble cargo, the symmetric variant of the KARM is suitable), is that fusion between two consecutive compartments occurs before the fission, which takes place in the area where thin tubules (or thinning of the proximal compartment) should be present. Thus, when the ERES is far away from the point of entrance into the Golgi, there should be a specific mechanism that ensures the main KARM postulate. Therefore, the KARM assumes that a tubule emanates from the CMC. This moves toward the ERES and hits this target. This tube might use kinesin for its movement along a microtubule. Indeed, the microtubule motor, kinesin, is present on membranes that cycle between the ER and the GC. At 37°C, kinesin was most concentrated on peripherally distributed ERES. The finding that kinesin is present on ERGIC structures is hard to reconcile with the VM, because the transport of carriers toward the GC is minus-end directed. Upon temperature reduction or nocodazole treatment, the kinesin distribution shifted onto the GC, while with brefeldin A treatment, kinesin is found in both Golgi-derived tubules and in the ER. This suggested that kinesin associates with membranes that constitutively cycle between the ER and the GC. The role of kinesin on these membranes was examined by microinjection of an anti-kinesin antibody. Golgi-to-ER, but not ER-to-Golgi, membrane transport was inhibited by the microinjected anti-kinesin antibody (Lippincott-Schwartz et al., 1995). Simultaneously, this peripherally moving tube delivers dynein to ERES for the consecutive centralization of the ER-Golgi carrier, which is formed within ERES. Such tubes have been described (Sciaky et al., 1997; Marra et al., 2007). This consequence of events explains why inhibition of kinesin blocks centralization of ER-Golgi carriers (Lippincott-Schwartz et al., 1995). On the other hand, Sec23p directly interacts with the dynactin complex. Co-localization of COPII and p150Glued was observed and turnover of Sec23 was increased after depolymerization of microtubules with nocodazole (Watson et al., 2005). This observation explains why in the absence of Sar1A and B (and as a consequence, in the absence of binding of Sec23p to ERES), nocodazole-dependent de-polymerization of microtubules does not induce fragmentation of the GC (Cutrona et al., 2013). Recently, Raote and Malhotra (2019) proposed that TANGO1 forms channels for procollagen that connect the ER and the GC.

Thus, the VM, DM, and CMPM cannot explain all of the data (i.e., prohibitive observations) that are contradictory to their logic, whereas the KARM should explain corner-stone observations that support the VM, DM, and CMPM.

## Intra-Golgi Transport

### The Vesicular Model

**Figure 5:**
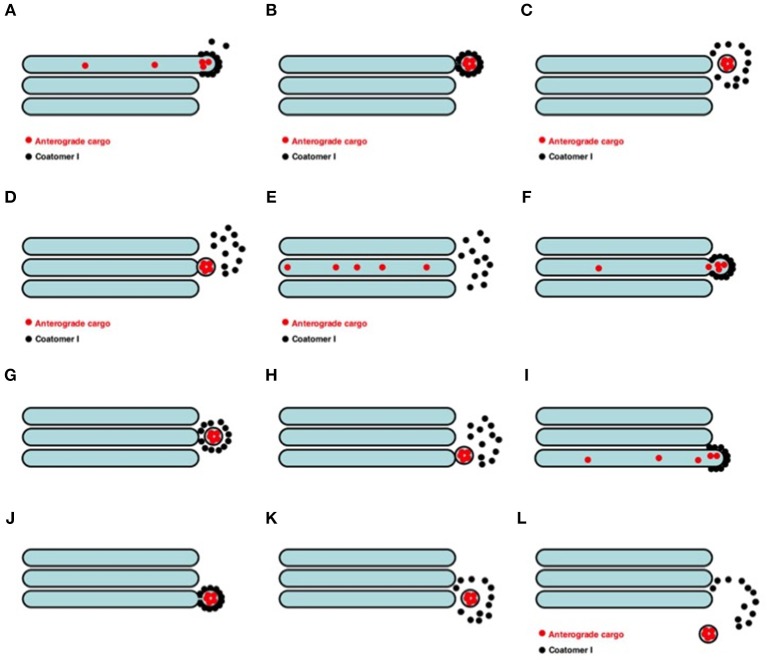
Scheme of the VM for intra-Golgi transport (see also movie https://yadi.sk/i/M1ykjxKabmjs5). **(A)** COPI (black dots) forms a coat on a membrane bud. A cargo (red dots) is concentrated inside the COPI-coated bud. **(B, C)** This bud undergoes detachment **(B)** and uncoating **(C)**. **(D)** The vesicle fuses with the distal Golgi compartment. **(E)** Distribution of the cargo within the next Golgi cisterna. **(F–H** and **I–L)** Repetition of the first stage. Finally, the vesicle can move out the Golgi **(L)**.

**Figure 6:**
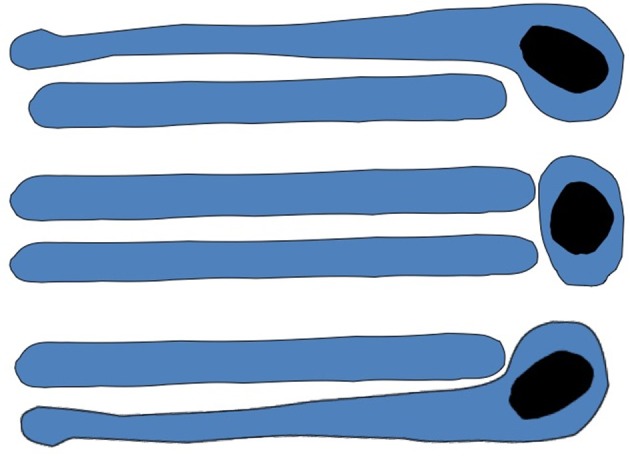
Scheme of megavesicle-based intra-Golgi transport. This hypothesis assumes that completely isolated megavesicle (in the middle of both images) can be found near the Golgi complex.

The main problem for the VM of IGT (Palade, 1975; Rothman et al., 1980, 1984) is the large cargo aggregates that are incompatible in size with COPI vesicles that cannot be transported by COPI vesicles (Figure 5; see also Mironov et al., 1997). The first evidence in favor of the existence of IGT of megacargoes in dynamic experiments was obtained by Bonfanti et al. (1998). The data by Becker et al. (1995) was presented not in the original paper but in a review. Moreover, it was then shown that this experimental model was incorrect (Perasso et al., 2000). The diameter of pre-chylomicrons is greater that the diameter of the internal volume of COPI-dependent vesicles. This also does not support the VM. Transport of VLDL particles through the GC of hepatocytes was demonstrated by Taylor et al. (1997). Transport of PCI through the GC was shown by Bonfanti et al. (1998). Transport of chylomicrons through the GC was suggested by Sabesin and Frase (1977). Similarly, secretory casein submicelles, which are transported through the GC in lactating mammary glands, are larger than COPI vesicles (Clermont et al., 1993). To solve this contradiction, the Rothman group proposed that such large cargoes are transported according to the CMPM, whereas VSVG is transported by vesicles (Orci et al., 2000b; Pelham and Rothman, 2000). Then, to adapt the VM to megacargoes (Figure 6), megavesicles were proposed (Volchuk et al., 2000; see their Figure 5). However, our analysis demonstrated that at the level of the GC, megavesicles do not exist (Mironov et al., 2001).

It is established now that the vast majority of cargoes are absent from COPI vesicles. We previously presented a list of cargo proteins that are excluded from COPI vesicles (Mironov et al., 2005). (Martinez-Menárguez et al., 2001) showed that the concentration of amylase in COPI-dependent vesicles is lower than in Golgi cisterna. Even Orci et al. (1986), the main proponents of VM, demonstrated that the VSVG aggregation inside 52–56 nm COPI-dependent vesicles was two-thirds of that in Golgi cisterna (see Table 1 of Orci et al., 1986). To support the VM, Orci et al. (1997) proposed that pro-insulin is transported by COPI vesicles. However, careful analysis of their study reveals that in Table 3 of Orci et al. (1997), the concentration of insulin in Golgi-associated round profile was one-third of that in the Golgi cisterna. To try to solve this contradiction, they proposed that there might be two populations of COPI vesicles: for anterograde and retrograde transport. According to (Martinez-Menárguez et al., 2001), amylase and chymotrypsinogen are excluded from the cisternal rims (and also from buds), whereas the KDEL receptor is not.

Furthermore, to provide additional support for the VM, the Rothman group (Pellett et al., 2013) transfected one population of cells with fluorescently tagged cargo tagged with one fluorophore, and another population of cells with Golgi-resident proteins tagged with another fluorophore. Next, heterokaryons were generated. These cargoes and enzymes were seen in small dots in the cytosol. A small portion of these particles contained COPI. Pellett et al. (2013) measured the diameters of these spots in the cytoplasm and isolated COPI vesicles from the cytosol using super-resolution light microscopy. Comparison of the diameters of these particles measured at the level of light microscopy with the diameter of isolated COPI vesicles, they concluded that these particles were COPI-dependent vesicles. They stated that the resolution of their super-resolution method is 80 nm. However, in reality (not in model experiments), resolution of stimulated emission depletion microscopy is about 100 nm (Sesorova et al., 2018). The real diameter of COPI vesicles is 52 nm (Marsh et al., 2001). This means that the resolution of their method is lower than the size of the structures they measured. The resolution of super-resolution microscopy depends on the refractory index of the medium, and *in-vitro* this parameter differs from that of cytosol. Also, the method used by Pellett et al. (2013) was very sensitive to the refractive indices of the media (Sesorova et al., 2018). Therefore, under these conditions, there could be significant systematic errors. Furthermore, they did not take into consideration that COPI-derived vesicles are on strings. This was discovered by Orci et al. (1998), and then confirmed by Marsh et al. (2001). Indeed, in mammalian cells, nobody has demonstrated coated or non-coated 52-nm vesicles at a distance of more than 200 nm (Martinez-Menarguez et al., 2001). Finally, it is known that diffusion of particles with a diameter of more than 50 nm is strongly restricted (Luby-Phelps, 1994).

Another problem with the interpretations presented by Pellett et al. (2013) is the volume-to-surface-area ratio of the transport carriers observed. According to Pellett et al. (2013), over 30 min, 25% of the membrane protein was transported from the GC of one cell to the GC of other cells. During this time, they observed 20,000 such particles. The rate of transport of soluble cargoes is identical to that of the membrane cargoes (see Figure 3 of Pellett et al., 2013). The diameter of COPI-dependent vesicles is 52 nm (Marsh et al., 2001). Their internal volume is 0.000045 μm^3^ and the surface area is 0.074 μm^2^. The Golgi volume is 1,500 μm^3^ (Mironov and Mironov, 1998). If we take into consideration that the ratio between the volume and the surface area of the GC is 140 (Ladinsky et al., 1999), the surface area of the GC would be 210,000 μm^2^. If these dots were COPI vesicles, 20,000 such vesicles would transport 1,480 μm^2^ of surface area (0.7% of the total), and 0.9 μm^3^ of Golgi volume (0.06% of the total). These considerations suggest that they observed the movement of carriers, that were much larger than COPI vesicles. Also in Figure 2f by Dunlop et al. (2017), the number of vesicles is not sufficient for IGT of soluble cargoes.

There are also other problems with the VM. There is a significant decrease in the number of COPI vesicles during synchronous IGT (Rambourg and Clermont, 1990; Rambourg et al., 1993; Fusella et al., 2013). Also in Figure 2f (by Dunlop et al., 2017), the number of vesicles is significantly lower than it is necessary for IGT of soluble cargo. There are no COPI vesicles in the microsporidia *Paranosema grylli* and *Paranosema locustae* (Beznoussenko et al., 2007), and very few in *Ostreococcus tauri* (Henderson et al., 2007), *Plasmodium falciparum* (Hohmann-Marriott et al., 2009) and *Tripanosoma cruzi* (see movies and Figure 2i of Girard-Dias et al., 2012). The VM cannot explain maturation of Golgi compartments in yeast in the absence of functional COPI (Matsuura-Tokita et al., 2006). Most anterograde cargoes are depleted in COPI vesicles (reviewed by Mironov et al., 2013). Albumin is depleted in COPI vesicles (Beznoussenko et al., 2014 see also Figure 7B of Dahan et al., 1994). S-Palmitoylation of anterograde cargoes at the Golgi membrane interface is an anterograde signal, and it results in the concentrating in curved regions at the Golgi rims, by simple physical chemistry (Ernst et al., 2018).

On the other hand, they did not observed vesicles on strings, which they had described earlier (Orci et al., 1998). The strings were considered as a mechanism that can prevent diffusion of COPI within the cytoplasm. The existence of megavesicles was not shown convincingly (an analysis of images of Volchuk et al., 2000, shows this). There is no COPI-like coat on the megabuds. There is no well-organized mechanism for fission.

### The Diffusion Model

There are several observations that favor the DM. To be relevant, the DM should be based on structures that are interconnected. Tubular connections between Golgi cisternae have been demonstrated by Marsh et al. (2004), Trucco et al. (2004), Beznusenko et al. (2006), and Bouchet-Marquis et al. (2008). Griffiths et al. (1994) described the bending of Golgi cisternae. Inter-cisternal connections are formed when a cargo arrives at the GC because Ca^2+^ is liberated and leaks from the Golgi compartments and the ER (Micaroni et al., 2010a,b); this leads to the fusion of COPI vesicles enriched in Qb SNAREs with Golgi cisternae, and the restoration of the Golgi SNARE complex. These connections between the Golgi cisternae are more abundant in transporting Golgi stacks and after stimulation of cell signaling (Clermont et al., 1994; Marsh et al., 2001; Trucco et al., 2004; Mironov and Beznoussenko, 2012; Mironov et al., 2017). These connections are permeable to albumin (Beznoussenko et al., 2014) and lipids (Pagano et al., 1989; Trucco et al., 2004). Moreover, dicumarol destabilizes Golgi tubules and delays IGT (Mironov et al., 2004), whereas after activation of protein kinase A, when the cisternae of the GC become interconnected, IGT is accelerated (Mavillard et al., 2010). This suggests an important role for these connections.

Some lipids can be easily transported along the secretory pathway when the formation of vesicles is inhibited (Sleight and Pagano, 1983; Pagano and Longmuir, 1985). In living cells, spots filled with fluorescent cargoes can move through the pre-bleached Golgi ribbon while they gradually lose their intensity (Presley et al., 1997). Finally, Patterson et al. (2008) reported that a cargo that exits the Golgi area shows exponential kinetics. This type of kinetics indicates that all of the compartments within the GC are interconnected. Patterson et al. (2008) also proposed that large cargoes that diffuse slowly might even exit from the *cis*-side of the GC. However, this last explanation is not valid, because PCI always exits from the *trans-*side of the GC (Bonfanti et al., 1998). Another problem of this study is the following: Patterson et al. (2008) did not examine the GC that is empty before the restoration of IGT. They examined only the GC that was already filled with cargoes. However, the process of Golgi filling with cargoes might take a significant amount of time, and under such conditions the exit kinetics might be different. The main problem of the DM is the protein, lipid and ionic gradients across the Golgi stacks, the presence of SNAREs within all steps of the secretory pathway, and the concentrating of cargoes (including megacargoes) during IGT. Megacargoes cannot diffuse along narrow intercisternal connections (Beznoussenko et al., 2014). Also, the concentrating of albumin (Beznoussenko et al., 2014) and large cargo aggregates that cannot diffuse along the intermediate compartment (Claude, 1970; Sabesin and Frase, 1977; Bonfanti et al., 1998; see below) do not support the DM (Mironov and Beznoussenko, 2008, 2012; Mironov et al., 2013).

### The Cisternal Maturation–Progression Model

**Figure 7:**
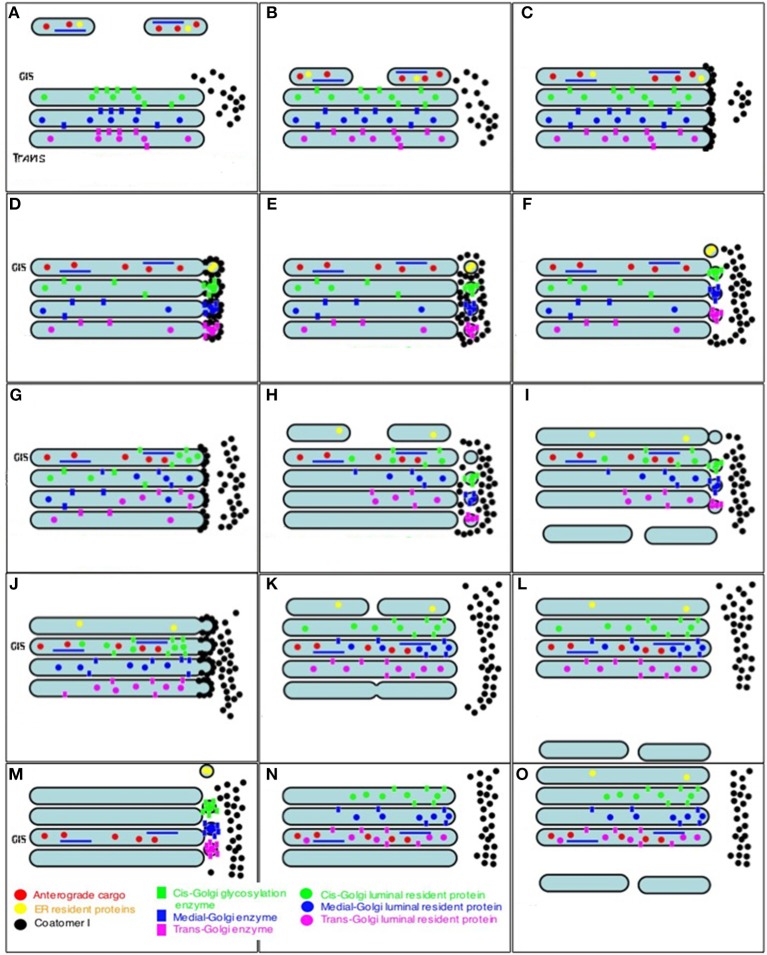
Scheme of intra-Golgi transport according to the CMPM. The main postulate of this model is that during intra-Golgi transport, the amount of cargo inside the cisterna during its progression is not changed, and that COPI vesicles (COPI, black dots; Golgi-resident proteins, colored dots) should be concentrated in COPI vesicles. **(A)** Formation of ER-to-Golgi carriers (top). **(B)** Delivery of ER-to-Golgi carrier to the Golgi complex. **(C)** Fusion of ER-to-Golgi carriers and formation of the new *cis-*Golgi cisterna. **(D)** Formation of COPI-(black dots) coated buds on the Golgi cisternae. **(E)** Detachment of buds and their uncoating. **(F)** COPI-dependent vesicles fuse with the proximal Golgi cisternae. **(G–I)** A new round of *cis-*cisterna formation, COPI-dependent budding, formation of vesicles, and their uncoating and fusion. **(I)** Departure of the most-trans cisterna in the form of post-Golgi carriers. **(J–N)** Additional rounds of similar events. **(O)** After step-wise departure of the post-Golgi carriers, the *cis-*Golgi cisterna formed after re-initiation of IGT becomes the *trans-*cisterna.

The cisternal maturation–progression model (Mironov et al., 1997, 1998b) poses that during IGT, each Golgi compartment undergoes maturation, by gradually transforming into the form of a more distal compartment as its resident proteins undergo recycling in COPI vesicles (Figure 7). At the level of the GC, the main prohibitive observation of the problems of the CMPM is the concentrating of soluble cargoes, regulated secretory cargoes and cargo aggregates during IGT (Oprins et al., 2001; Mironov and Arvan, 2008; Beznoussenko et al., 2014). We have reported the concentrating of albumin during IGT (Beznoussenko et al., 2014). Therefore, we proposed that two different mechanisms of IGT could function simultaneously; namely, one for albumin and a1-antitrypsin, and another for PCI (Beznoussenko et al., 2014). Also, Oprins et al. (2001) demonstrated the concentrating of regulatory secretion cargoes during their journey through the GC, before their precipitation within secretory granules. Further, the concentrating within megacargoes is evident from images presented in different studies.

We demonstrated the concentrating of albumin (Beznoussenko et al., 2014), but to be politically correct, we need to explain this discrepancy on the assumption that PCI and albumin use different modes of IGT. McCaughey et al. (2019) also observed the concentrating of a specifically designed fusion protein based on procollagen in the last medial Golgi compartment (their Figure 1). Although they did not determine whether their chimeric protein behaved exactly as the natural procollagen, their observations with this cargo protein contradicts to the main prohibitive observation of the CMPM. In any case, stably transfected cells did not show any sign of accumulation of their protein within the Golgi area after the release of the RUSH-dependent procollagen from the ER. The concentrating during IGT has not only been shown for albumin (Beznoussenko et al., 2014) and the regulated secretory proteins (Oprins et al., 2001; Mironov and Arvan, 2008), but also for the megacargoes PCI (see Figure 4B of Bonfanti et al., 1998), chylomicrons and VLDL (see above).

The main prohibitive observation for the CMPM is the concentrating of cargo during intra-Golgi transport. We have shown that albumin is concentrated during IGT (Beznoussenko et al., 2014). The concentrating of PCI in cisternal distensions was shown by Bonfanti et al. (1998 see their Figure 4). The concentrating of chylomicrons in cisterna distensions at the *trans-*side of the GC in enterocytes was shown by Sabesin and Frase (1977). The concentrating of VLDL in cisternal distensions of the GC in hepatocytes was shown by Claude (1970). The concentrating of lipid particles in cisterna distensions at the *trans-*side of the GC was also demonstrated by Glaumann et al. (1975) (their Figure 7b) and Matsuura and Tashiro (1979) (their Figures 1, 9, 14). During IGT, the number of chylomicrons in one cisternal distension increased (Sabesin and Frase, 1977). This does not support the CMPM (Mironov et al., 2013). Also, in Figures 1, 6 of Dahan et al. (1994) the levels of Apo E, a marker of lipid particles, were seen to increase at the *trans-*side of the GC, in comparison to the *cis-*side. The concentrating of large cargo aggregates that cannot diffuse along intercompartmental connection does not support the CMPM, and also does not support the DM. According to the CMPM, different cargoes should not move across the Golgi stacks with different speeds in the *cis*-to-*trans* direction. However, soluble cargoes reached the trans-side of the Golgi faster than VSVG and procollagen (Beznoussenko et al., 2014). Also aggregates of cargo proteins inside cisternal distensions of Golgi cisterna moved faster than the specific cisterna domains where the opposing membranes were connected by protein bridges (Lavieu et al., 2013).

The problem of the concentrating of Golgi-resident proteins in COPI-dependent vesicles is very serious (Glick et al., 1997). Golgi-resident proteins should not be depleted in COPI-dependent 52-nm vesicles, because if the concentration of these proteins in the vesicles were lower than in the Golgi cisterna, the recycling of these proteins would be very slow. Mathematical modeling based on the CMPM assumptions demonstrated that significant concentrating of the Golgi enzymes in the vesicles is necessary for the CMPM (Glick et al., 1997). Importantly, significant depletion of Golgi-resident proteins in COPI vesicles was discovered in *S. cerevisiae* (Beznoussenko et al., 2016). Depletion of Golgi-resident proteins in COPI vesicles (Volchuk et al., 2000; Kweon et al., 2004; Gilchrist et al., 2006; Fusella et al., 2013; Beznoussenko et al., 2016) would not support the CMPM. However, Golgi glycosylation enzymes (Velasco et al., 1993; Cosson et al., 2002; Kweon et al., 2004), nucleotide sugar transporters (Fusella et al., 2013), syntaxin 5 (Orci et al., 2000a,b; Gilchrist et al., 2006), p24, ERGIC58, TGN38/46 (Gilchrist et al., 2006) are depleted in COPI vesicles. In spite of this, several groups have reported the concentrating of some Golgi-resident proteins in COPI vesicles. A single vesicle filled with the Golgi enzyme galactosyltransferase was shown in the study of Grabenbauer et al. (2005). However, the DAB reaction is not quantitative, and it is not possible to judge whether the concentration of galactosyltransferase in the COPI vesicle was higher or lower than in the corresponding cisterna. Gilchrist et al. (2006) demonstrated that the concentration of Golgi enzymes in the light membrane fraction obtained after incubation of isolated Golgi membranes with cytosol and GTP is higher than in the Golgi cisternae, whereas anterograde cargoes were depleted there. Electron microscopy has revealed that this fraction is composed of 52-nm vesicles. Gilchrist et al. (2006) concluded that COPI-dependent vesicles are retrograde transport carriers for the Golgi enzymes. However, careful analysis of their data revealed that when the light fraction was prepared for electron microscopy, it was additionally pelleted onto a sucrose cushion (50% [w/w]) at 45,000 rpm. This procedure was not used for the biochemical measurement of the Golgi enzyme concentrations in this fraction. This sucrose-based centrifugation was also not used in their previous study (Lanoix et al., 1999), and the purity of these 52-nm vesicles was significantly lower. Therefore, it could be proposed that in their light fraction, there were perforated fragments of Golgi cisternae enriched in Golgi enzymes (Kweon et al., 2004), whereas after the additional centrifugation, only 52-nm vesicles remained in the samples prepared for electron microscopy. This might explain why the concentrations of the enzymes in the light fraction were higher than in the isolated Golgi membranes. However, in the same study, several other resident Golgi proteins had lower concentrations in the light fraction than in the isolated GC (Gilchrist et al., 2006).

Further, (Martinez-Menárguez et al., 2001) revealed that *in situ*, mannosidase II is 1.6-fold more concentrated in COPI-coated peri-Golgi round profiles. However, these data do not demonstrate that this Golgi enzyme was enriched in really separated COPI vesicles because according to the quick-freezing data of Marsh et al. (2001), the vast majority of 52-nm vesicles are not coated. Moreover, on cryosections, it is not possible to distinguish a section of a COPI vesicle from a section of a COPI-coated tube, and sections of tangential tubules can give 52-nm round profiles coated with COPI. Indeed, it was demonstrated that tangential tubules are coated with COPI (Weidman et al., 1993; Yang et al., 2011). Importantly, the vast majority of the 52-nm vesicles within the Golgi area are not coated (Marsh et al., 2001). Moreover, Cosson et al. (2002) showed depletion of this enzyme in these round profiles.

Recent observations that demonstrate that mannosidase I can be recycled by COPI vesicles are not convincing (Rizzo et al., 2013). Rizzo et al. (2013) demonstrated that after the polymerization of the chimeric mannosidase I (ManI-FM), this protein shifted from the *cis-*side to the *trans-*side of the GC. Although the monomer form of this chimera is depleted in near-Golgi round profiles, after the depolymerization, ManI-FM quickly appeared in round profiles. According to this study, 50% of these round profiles were coated with COPI. These data were interpreted in favor of the CMPM. However, Rizzo et al. (2013) did not use serial cryosections to distinguish between the round profiles as projections of cross-sections of tubules and the projections of actual vesicles. On random cryosections, this distinction is not possible (Kweon et al., 2004). Moreover, on cryosections, a tubular network can appear as round profiles, which were considered in this study as COPI-dependent vesicles. Also, Rizzo et al. (2013) did not perform the obvious control experiment based on inhibition of the formation of vesicles by COPI (i.e., microinjection of cells with an antibody against ßCOP). Also, their tomography data presented in favor of the augmentation of the number of COPI-dependent vesicles after the depolymerization are not convincing.

Indeed, they stated, “All reconstructions indicated that the morphology of the carriers and the structure and size of the Golgi stack were similar under all experimental conditions. Moreover, tomography confirmed that most round, 50- to 80-nm structures were indeed vesicles, and that the relative frequency of vesicles and tubules was similar to that seen in thin sections (not depicted).” To demonstrate that round profiles represented separated vesicles, they showed very small sized serial electron microscopy tomography images of only one round profile. However, this round profile showed a visible neck that connected it with a Golgi cisterna. This neck is visible on frames 45–55 of Rizzo et al. (2013). If we take into consideration that the thickness of their tomography slice was 3 nm and the resolution of the presented the images is 10 nm, the obvious conclusion is that this neck represents a membranous structure, and that this round profile actually represents a COPI-coated bud. The statement that 50% of the round profiles were coated with COPI also favors our explanation because a vast majority of free vesicles near the GC are not coated (Marsh et al., 2001). No electron microscopy tomography images that showed the effects of the ManI-FM depolymerization were presented (see the analysis of the paper by Rizzo et al., 2013, in the Supplementary Materials). Thus, these data that show the concentrating of the Golgi-resident proteins in COPI vesicle are not convincing.

During synchronous IGT, the number of COPI vesicles should be sufficient for the recycling of all of the resident proteins. However, when a large amount of cargo moves across the GC, even if we take into consideration the maximal possible speed of COPI vesicle formation (Mironov et al., 2001; Trucco et al., 2004; Fusella et al., 2013), the number of COPI vesicles is less than one-tenth of that necessary for the transport of membranes across the GC. Thus, even when COPI vesicles were generated at maximal speed, the rate of their generation can support only 10% of the vesicles necessary for IGT (Fusella et al., 2013).

Sialyltransferases and fucosyltransferases are present within the *trans-*most cisterna (TMC). However, there are no buds coated with COPI on the TMC (Ladinsky et al., 1999; Marsh et al., 2001; Mironov et al., 2017). In principle, it is possible that clathrin-dependent vesicles can function as retrograde carriers. Indeed, Velasco et al. (1993) observed labeling of mannosidase II with DAB in clathrin-coated buds within the GC. However, DAB labeling is not quantitative. Moreover, Rothman et al. (1980) reported that the clathrin-dependent vesicles isolated from the GC contained not Golgi-resident proteins, but the cargoes. Ladinsky et al. (1999) and Grabenbauer et al. (2005) showed that within the Golgi area there were only eight clathrin-coated vesicles. This is the diameter of COPI-dependent vesicles. It is important to underline that the diameter of COPI-dependent vesicles is very uniform (Marsh et al., 2001). If we take into consideration that the surface area of these Golgi clathrin-dependent vesicles is only 1.3-fold higher than that of COPI-dependent vesicles, the obvious suggestion is that the number of clathrin-dependent vesicle is about one twentieth of the number necessary for synchronous recycling of the Golgi-resident proteins.

Moreover, although there have been several statements that COPI-coated buds can be found within the TMC, or even the TGN, in reality this has not been completely established. There was no convincing evidence in the images that (Martínez-Menárguez et al., 1999) provided that these structures were really TMC/TGN. For instance, in Figure 5B of (Martinez-Menárguez et al., 2001), the coated bud labeled for ßCOP is localized at the *trans*-side of the Golgi. However, there is no clear membrane continuity of this bud with the TMC or with structures of the TGN. Thus, this image might be the section of a COPI-coated bud of the medial cisterna, and this might be an effect of the section plane. Its diameter is 60 nm. This diameter is too high for COPI vesicles. Figure 5C of (Martínez-Menárguez et al., 1999) shows a membrane bud with a protein coat on the immature secretory granule within the GC of an acinar epithelial cell from the rat pancreas. However, this granule is immature and might represent a distension of the last medial cisterna. Examples of such distensions of the medial Golgi cisternae with no completely dense content can be easily found in images presented on the website “nanotomy.” Moreover, we also detected COPI-coated buds on the cargo domains, which appeared on the distension of the TMC, but only during cargo synchronization according to the maxi-wave protocol, when a large amount of cargo moves simultaneously through the GC. Thus, everything should be considered in terms of probability, and the probability of finding COPI-coated buds on the TMC is relatively low. As such, it is not clear how the resident proteins undergo recycling from the TMC. The variability of the diameters of the COPI vesicles described by Martínez-Menárguez et al. (1996) might be a result of chemical fixation or of the methods of measurement. At least in our hands, when we inhibited SNAREs and most of the vesicles formed within the Golgi area derived from the activity of the COPI machine, their diameters were extremely uniform (Kweon et al., 2004; Fusella et al., 2013).

The third prohibitive observation for the CMPM is the situation when renovation and progression of Golgi cisternae can be blocked. However, under these conditions IGT was observed although it became slower (Dunlop et al., 2017). Also when the opposite membranes of the megacargo domain are connected by protein bridges, its IGT transport was inhibited (Lavieu et al., 2013). Moreover, “the land-locking” of Golgi cisternae does not exclude the possibility that the KARM might also explain these data. Indeed, Golgi cisternae visible within the mitochondria aggregates were relatively close to each other, and could be temporally connected by tubules.

The CMPM has several other problems. The full list of the CMPM problems was presented in our previous review (Mironov et al., 2013). In microsporidia, there are no COPI vesicles for the recycling of resident Golgi proteins (Beznoussenko et al., 2007), although IGT takes place. Also, the study of Patterson et al. (2008) does not support the CMPM, because within the framework of the CMPM, the exit of PCI-GFP from the Golgi zone after bleaching of the whole cell less the Golgi area should not be negatively exponential. This should be composed of two parts to the regression line. The first part should be horizontal, and the second part should be as for linear decay. Another explanation that they presented is the proposal that PCI dots can exit from the GC immediately after their arrival at the *cis-*side of the Golgi stack, without their progression across the stack. Their third explanation suggests that megacargoes might diffuse along the lumen of the united membrane system of the Golgi stack. Finally, the CMPM cannot explain the observation that shows that overexpression of the GDP-mannose transporter in the yeast *S. cerevisiae* induces the formation of stacked Golgi (Hashimoto et al., 2002). It is not clear how the IGT is organized under these conditions, because the main adaptation of the CMPM for *S. cerevisiae* is that due to the spatial separation of the different Golgi compartments, there should be a mechanism for the directionality of the delivery of COPI-dependent vesicles. On the other hand, Rambourg et al. (1993) observed that in sec7 mutants maintained at 37°C in low (0.1%) glucose medium, secretion granules progressively decreased in number, and soon disappeared. Concomitant to this, the networks of Golgi tubules increased in size and complexity, lost their distensions, and then transformed into flattened saccules that formed stacks of up to seven or eight saccules that were similar to the Golgi stacks seen in mammalian cells. Indeed, if we remember that in *S. cerevisiae*, the different Golgi compartments should have contact with each other to fulfill the transport of cargo from one compartment to another.

### The Kiss-and-Run Model

**Figure 8:**
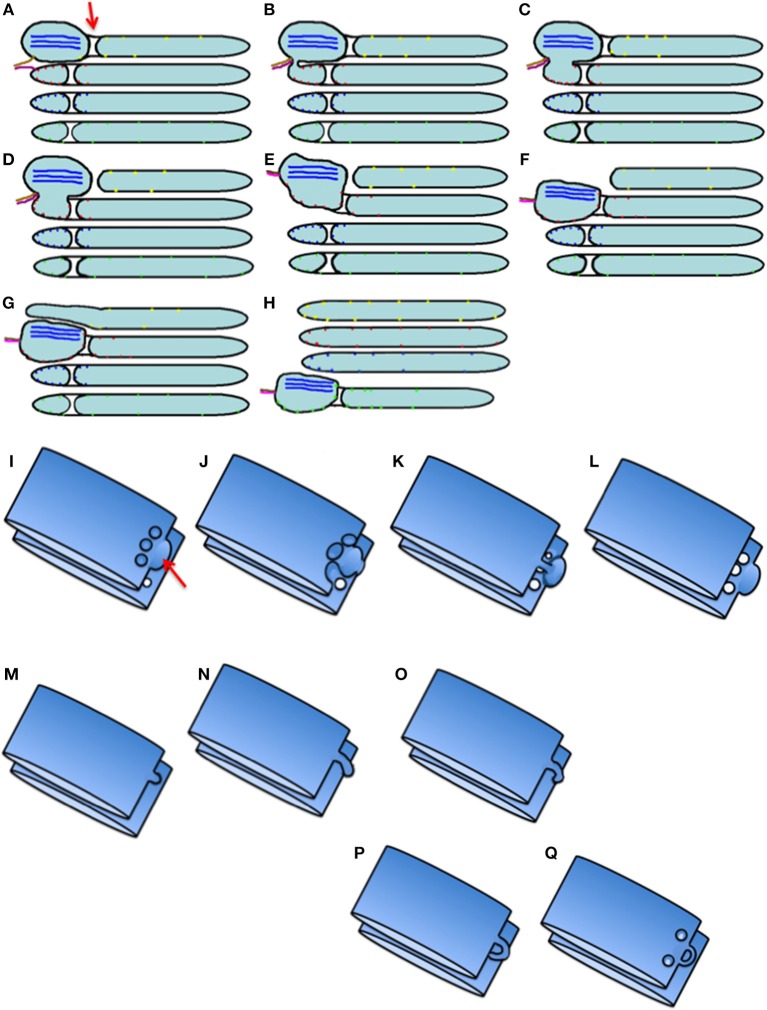
Scheme of intra-Golgi transport according to the KARM. **(A–H)** Its main principle is the following: initially there is SNARE-dependent fusion with the distal Golgi cisterna, and then fission along the line of the pore row. **(I–Q)** Scheme showing only two cisternae in three-dimensions. **(I)** Formation of the cargo domain (red arrow) separated by the row of pores. **(J)** Fusion the domain with the distal cisterna and enlargement of pores. **(K)** Break down of two tubules surrounding pores. **(L)** Break down of the last tubule connecting the domain with the proximal cisterna. **(M–Q)** Stages of pore formation, when membrane bud grows and then fuses backward to the same cisterna. During the transport wave, there is consumption of the cisternal pores. Images similar to Figure 8B can be found in Trucco et al. (2004) (see their Figures 3l–n).

To be efficient, the KARM of IGT should be based on several prerequisites (Figure 8; see above). Some of them were already observed. Indeed, cisternal distensions containing megacargoes already represent cargo domains. The VSVG domains that do not exchange this cargo with each other were described by us (Mironov et al., 2001). We demonstrated how the KARM explains IGT of soluble cargoes and Golgi glycosylation enzymes (Mironov and Beznoussenko, 2012; Mironov et al., 2013). On cryosections, cisternal pores are not usually particularly visible. However, even in Figure 1 of Bonfanti et al. (1998) the pores were visible. In Figure 2 of Bonfanti et al. (1998), the pores are not particularly visible due to the diffusion of the DAB. The excessive diffusion of the DAB precipitate hid these pores. In Figures 6E,F of Mironov et al. (2017), pores can be seen between the PCI-containing distensions and the rest of the cisternae. In Figure 3 of Mironov and Pavelka (2008), pores are visible as well. Cisternal distensions in the GC of acinar pancreatic cells are separated from the rest of cisternae by rows of pores. This was visible in the studies of Claude (1970), Sabesin and Frase (1977), and Sesso et al. (1994). Initially, albumin is present in cisternal distensions that are filled with VLDL (Figure 6e,f of Dahan et al., 1994). However, these pores appear to be very important for IGT (Figure 8).

Pores that separate cisternal distensions from the rest of the Golgi cisternae were shown by Claude (1970) in hepatocytes; by Sabesin and Frase (1977) and by Pavelka and Roth (2005) (their Figure 101A, page 205) in enterocytes, where the GC transports chylomicrons; by Sesso et al. (1994) (their Figures 5, 7a–d, 9, 10) in acinar pancreatic cells; by Ladinsky et al. (2002) after the 20°C temperature block; by Mironov et al. (2001) (their Figures 4C,E,F) in fibroblasts transporting PCI aggregates; by Pavelka and Roth (2005) (their Figure 98B, p. 199) in hepatocytes during IGT of VLDL. In Figures 6E,F of Mironov et al. (2017), which shows the GC in enterocytes, the pores can be seen between the cisternal distensions filled with chylomicrons and the other parts of the Golgi cisterna.

Thus, restoration of pores in the cisternal rims might be based on this mechanism. The KARM gives the following predictions: (1) if pores inside cisternae are consumed, there should be the need for resting of the Golgi stack; (2) recycling of Ykt6 is improbable. As such, there should be one use of this SNARE, and after consumption of the cytosolic pool of Ykt6, there should be the need for the resting of the whole Golgi complex in the entire cell. Thus, there could be several waves of cargo, and only then would the pores be consumed. Fission and then fusion of COPI vesicles might induce the formation of tubules and restoration of the number of pores along cisternal rims (Park et al., 2015).

Careful analysis of images presented by Ladinsky et al. (2002) and Taylor et al. (1997) revealed that after 2 h of the 20Â°C temperature block in the presence of inhibitor of protein synthesis, when several waves of cargo protein (VSVG) passed through the GC, the numeric density of pores in Golgi cisternae decreased (Figures 3C–F, 4 by Ladinsky et al., 2002). In contrast, after prolonged inhibition of IGT, this density increased (see Figure 3 by Taylor et al., 1997).

When membranes are transported through the GC, the asymmetric variant of the KARM should be used. According to this, to increase the efficiency of IGT, there should be cargo domains where a cargo is concentrated. These domains should contain a set of SNAREs complementary to those in COPI vesicles (GS27, GOS28) (Fusella et al., 2013), and be somehow separated from the rest of the Golgi cisterna, to facilitate the fission process. Finally, during IGT, all of the cargoes, including large cargo aggregates, should undergo concentrating at the *trans-*side of the GC. In contrast, according to the CMPM, these first three demands are not necessary, whereas, the fourth is forbidden. Good examples of such cargo domains might be: PCI-containing distensions of Golgi cisternae in collagen-secreting cells (i.e., fibroblasts); chylomicron-containing distensions formed during transcytosis of lipids through enterocytes; and cisternal distensions filled with VLDL particles in hepatocytes. The presence of a row of pores behind the cargo domain during IGT and the concentrating of SNAREs over the cargo domain favor the KARM. Our observation that there is no cargo diffusion between VSVG-GFP domains formed during IGT (Mironov et al., 2001) is in favor of the KARM.

## Discussion

Thus, at the level of ER-Golgi and IGT the VM faces with the problem of the transport of megacargoes. Attempts to modify the VM by addition of so called megabuds and megavesicles were not convincing. We suggest that the megavesicles observed by Gorur et al. (2017) and the large procollagen-positive immobile dots observed by Omari et al. (2018) and McCaughey et al. (2019) (their Figure 2) represent ER-derived autophagosomes (reticulophagosomes; Fregno and Molinari, 2018; Fregno et al., 2018; Omari et al., 2018; Forrester et al., 2019). Autophagosomes derived from the protrusions of the ER filled with protein aggregates were first described by Omari et al. (2018), (Fregno and Molinari, 2018 see also Fregno and Molinari, 2018; Forrester et al., 2019). In a study of Fregno et al. (2018), this phenomenon was observed upon overexpression of the secretory heavy chain of immunoglobulin M that lacked some domains; the aggregates of this chain were concentrated in ER protrusions with the diameter of ≥450 nm. These protrusions were not coated with a COPII-like coat. After detachment from the ER, these distensions were delivered to the GC, and then were secreted or fused with lysosomes (see SM).

CMPM has problems at both steps of intracellular transport, namely, at the level of the exit from the ER, it cannot explain the problem of different concentration of different cargoes whereas at the Golgi level, it cannot explain concentration of megacagoes during IGT. Although when we faced similar discrepancy between the *cis-to-trans* delivery of albumin and PC we tried to combine different models for different cargoes and used the DM for the explanation of this delivery. However, the DM cannot explain the augmentation of albumin concentration at the trans side of the GC. The second main problem of CMPM for IGT is the depletion of several resident proteins in the so called retrograde COPI vesicles. Moreover, the recycling at the level of the trans-most cisterna and the TGN is not possible to explain because COPI vesicles which are considered to be retrograde transport carriers are not formed. The attempt to propose that clathrin-dependent vesicles could execute the recycling of the resident proteins is not successful also due to the absence (or extreme rarity) of COPI-coated buds on the trans-most cisterna (see details in the Supplementary Materials).

**Figure 9:**
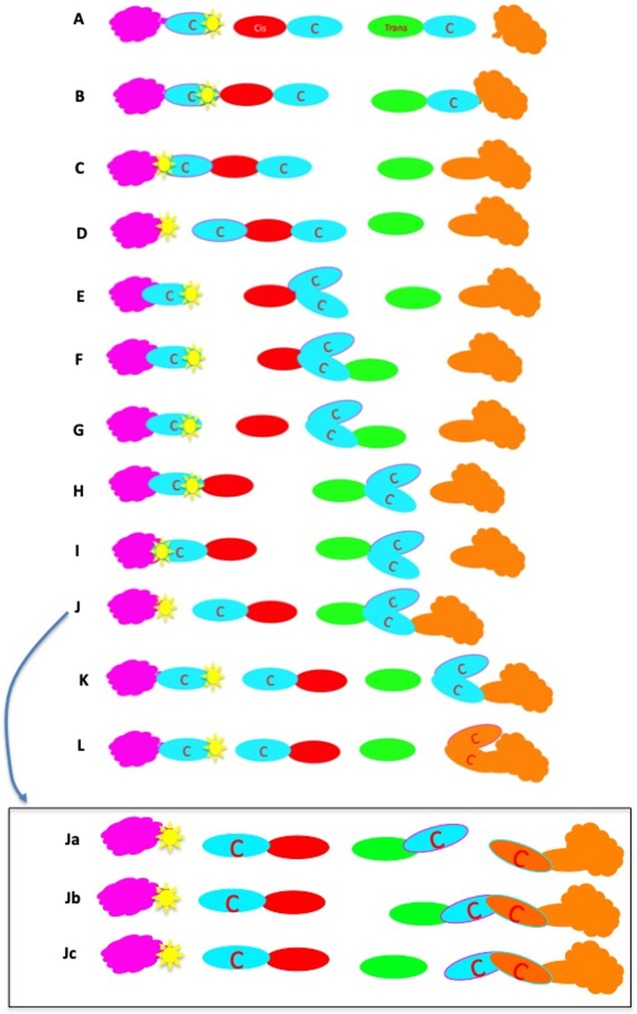
Scheme of intra-Golgi transport in ***S. cerevisiae***within the framework of the KARM. This scheme ensures maturation and progression of cargo domains. According to the main postulate of the KARM, to become separated, it is necessary to have initial fusion. Thus, fusion is the first event, and then fission (at another site) is the second event. The ER is magenta; artificial cargo is blue and indicated with the letter “C”; the *cis-*Golgi compartment is red; the trans Golgi compartment is green; the post-Golgi compartment is brownish; the exited cargo domain is surrounded with a red line. The situation that is shown here was formed after several rounds of cargo domain detachment from ERES, when most of the Golgi compartments already contain the artificial cargo. **(A)** The initial situation when most of the Golgi compartments contain cargo domains (blue). **(B)** Fusion of the *cis-*Golgi compartment (red) with ERES, and the *trans-*Golgi compartment (green) with the post-Golgi compartment (brownish). **(C)** Shift of the COPII coat (yellow) from the cargo domain (blue oval surrounded by a magenta ring). There is fission of the connections between the trans compartment (green) and the cargo domain, which is consumed by the post-Golgi compartment. **(D)** Detachment of the *cis-*Golgi compartment connected to two cargo domains from ERES (yellow). **(E)** Rotation of the *cis-*Golgi (for the sake of clarity). **(F)** Fusion between the *trans-*Golgi compartment and the *cis-*Golgi compartment connected to cargo domains. **(G)** Detachment after fission of the *cis*-Golgi compartment from the *trans-*Golgi compartment connected to cargo domains. **(H)** Fusion of the *cis-*Golgi compartment with ERES, where a new cargo domain covered with Sec31 is prepared. **(I)** Shift of the Sec31-coat (yellow) from the cargo. **(J)** Detachment of the *cis-*Golgi compartment connected with the new cargo domain from ERES, and fusion of the *trans-*Golgi part connected to cargo domains with the post-Golgi. **(K)** Detachment of the *trans-*Golgi compartment from the cargo domains. **(L)** The final stage when the cargo domain of interest is within the post-Golgi compartment. **(Ja–c)** The alternative consequence of the final events; namely, the cargo domains connected with the *trans-*Golgi compartment can be delivered to the post-Golgi one after another.

The DM cannot explain the necessity of SNAREs for intracellular transport and concentration of cargo at different level of the transport. Thus, the VM, DM, and CMPM cannot overcome their prohibitive observations. Also the VM, DM, and CMPM cannot explain the mechanisms of Golgi ribbon formation and the disappearance of the GC in *S. cerevisiae*, the fragmentation of the Golgi ribbon In contrast, the KARM can do this. The live-cell imaging of RUSH-controlled cargoes shows that different cargoes have different kinetics (Boncompain et al., 2012). One possibility is that they follow different trafficking mechanisms; another one is that rates of different cargo concentration are different. The studies usually considered as the corner stones of the VM, DM, and CMPM could also be easily explained from the point of view of the KARM. Although we have shown here that now the KARM appears to be the most powerful model of IGT, it still has some difficulties. For instance, one of these is the existence of separate and different Golgi compartments in *S. cerevisiae*. The observation that different Golgi compartments are rarely connected by tubules might provide the explanation, and thus the requirements of the KARM (Beznoussenko et al., 2016). Moreover, Kurokawa et al. (2019) demonstrated that Golgi-resident proteins and a cargo can form two domains within the same Golgi compartment. In *S. cerevisiae*, the different Golgi compartments are separated. If we assume that in *S. cerevisiae* IGT occurs according to the CMPM, the mechanism of the vectorial delivery of retrograde COPI vesicles should exist. For instance, in mammalian cells, COPI vesicles are on strings. This prevents the diffusion of COPI vesicles around the GC, and might explain the vectoriality of vesicle movement (Orci et al., 1998). However, proponents of the CMPM have not presented any analysis of this problem in *S. cerevisiae*. When this review was already submitted, two important studies appeared (Casler et al., 2019; Kurokawa et al., 2019). In both of these, visualization of maturation of the cargo domain was performed in living *S. cerevisiae* cells. The authors claim that their studies demonstrate the cisterna maturation model. However, their data fit even better to the KARM (Figure 9).

The vesicle delivery is a very important problem for both VM and CMPM and especially for *Saccharomyces cerevisiae*. The diffusion of vesicles through the dense cytosol is very slow, because most of these vesicular carriers have a diameter >50 nm (Luby-Phelps, 1994). In order to solve this, the idea of “vesicles on a string” was proposed (Orci et al., 1998). On the other hand, different Golgi compartments could be getting closer. When these compartments are close by, a burst of COPI vesicle can be formed to provide the transfer of a significant amount of protein from one compartment to another. However, in this case the number of COPI-coated buds on the Golgi compartments should increase. However, this number is much lower than in mammalian cells (Beznoussenko et al., 2016).

**Figure 10:**
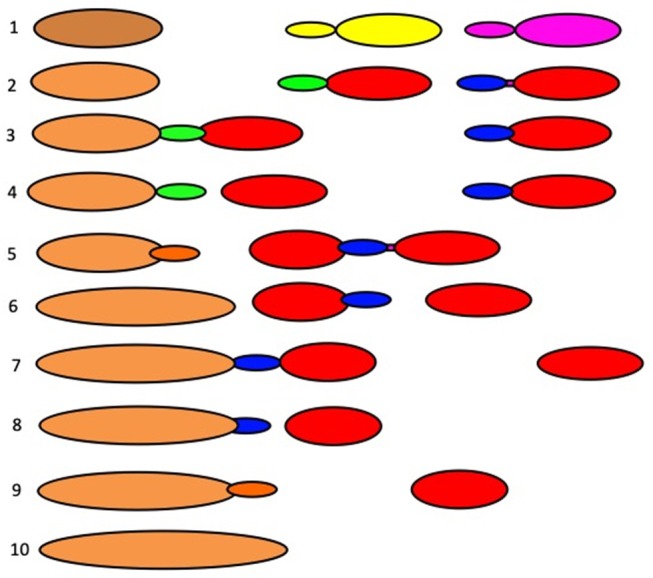
The scheme explains why the Golgi complex disappears after block of cargo exit from the ER in *S. cerevisiae*. The postulates are the same as in Figure 9.

The CMPM cannot explain the observation that demonstrates that in *S. cerevisiae* when cargo exit from the ER is blocked, the GC disappeared (Ayscough and Warren, 1994; Morin-Ganet et al., 2000). The KARM can do this (Figure 10). Also, the CMPM cannot explain why after elimination of COPI vesicles using the temperature sensitive mutant of one of the COPI subunits (Matsuura-Tokita et al., 2006), compartment maturation continues, although it becomes slower. In contrast, the KARM posing that cisternal pores are important and are consumed during IGT explains this phenomenon easily. Also we presented the explanation of the corner-stone experiments, which are usually considered in favor of the VM and CMPM. This explanation fits to the KARM.

The important issue, which the KARM should explain, is the role of COPI and COPI-dependent vesicles. Within the framework of the KARM, COPI vesicles are important for: (1) elimination of excessive membrane curvature (Beznoussenko et al., 2015); (2) extraction of Qb SNAREs and slowing down of IGT (Trucco et al., 2004; Fusella et al., 2013; Beznoussenko et al., 2016); and (3) initial retention of Golgi enzymes (Dominguez et al., 1998; Nilsson et al., 2009). Park et al. (2015) showed that COPI can sort anterograde cargoes into COPI-dependent tubules. Importantly, tubulation of the GC accelerates EGT and IGT (Mavillard et al., 2010; Capaci et al., 2019). Membrane coats are necessary for the concentrating of SNAREs (Zeuschner et al., 2006; Pryor et al., 2008; Koo et al., 2011; Fusella et al., 2013). However, it is necessary to test whether the transport of cargoes would be stopped when inhibition of the specific set of SNAREs localized at defined steps of intracellular transport would stop the transport at this specific point. The discovery of such mechanisms, or their absence, might confirm or reject the KARM. The additional role of COPI-dependent vesicles could be their involvement in the uncoating of Golgi membranes from COPI coat.

There are two transport steps where this scheme might be not very obvious; namely, EGT and post-Golgi transport. There, according to the KARM, the tubule from the GC has to move toward the ER exit site (ERES), and this should induce a bolus-like delivery of ER-to-Golgi carriers. At the post-Golgi stage, the tubule or endosome *per se* has to move toward the centrally localized GC. After the fission, the cargo domain should move centrifugally according to the bolus-like mechanism and thin tubules behind the cargo domain could be observed (Polishchuk et al., 2003). When compartments are separated by significant space the distal compartment moves toward the proximal one, fuses with this cargo domain and traps the cargo domain, exactly as was shown by (Casler et al., 2019 see below). The tubules formed by COPI could fuse with the cargo domain localized within the proximal Golgi domain, as was shown by (Trucco et al., 2004 see Figures 3i–n there). The concentrating of SNAREs at the future fusion sites increases the efficiency of transport, which eliminates stochastic events. Also it is not necessary to have separate retrograde carriers of separate retrograde pathways. During the kiss-and-run process there could be a simultaneous process of anterograde and retrograde exchange between the two compartments. Thus, the KARM has a significant potential for the role of a new paradigm within the transport field. Nevertheless, additional analysis of this issue is necessary.

Thus, the KARM give the following predictions. (1) The cargo should be organized in the domain more or less clearly separated from the domains, which are formed by Golgi-resident proteins. (2) Initially, there should be lateral contact between the cargo domain and the Golgi domain. Then the cargo has to go together with the Golgi compartment. (3) After its arrival at ERES, the *cis-*compartment displaces Sec31 from the cargo domain. (4) After arrival of the *cis-*compartment at ERES, the size of the cargo domain should increase (**C**). (5) The transient phase of maturation when domains of two different Golgi compartments are situated within the same Golgi cisterna should be shorter than other (co-localization) phases when only one Golgi compartments is present within the same Golgi cisternae (This explains why in yeast, intermediate forms of transport carriers are rare). (6) The cargo should be concentrated into two cargo domains. However, if other cargoes (which are invisible to the observer) are transported simultaneously, this multiplication should be slightly lower than 2-fold. (7) During the transitional phase, the initial co-localization would be between cargo and trans and not between cis and trans markers. (8) The cargo-domain and the Golgi domain should be in the same membrane disk with pores, but within their own domains. The cargo domains should be separated from the rest of the Golgi cisterna by pores.

## Author Contributions

All authors listed have made a substantial, direct and intellectual contribution to the work, and approved it for publication.

### Conflict of Interest Statement

The authors declare that the research was conducted in the absence of any commercial or financial relationships that could be construed as a potential conflict of interest.

## Abbreviations

CMPM: compartment (cisterna) maturation–progression model
COP: coatomer
DM: diffusion model
EGT: ER-to-Golgi transport
ER: endoplasmic reticulum
ERES: ER exit site
GC: Golgi complex
IGT: intra-Golgi transport
KARM: kiss-and-run model
PCI: procollagen I
VLDL: very low-density lipoprotein
VM: vesicular model
VSVG: G protein of the VSV virus.
