# Supplementary material for: Models of Intracellular Transport: Pros and Cons
Source: Front Cell Dev Biol. 2019 Aug 7;7:146. doi: 10.3389/fcell.2019.00146 (PMC6693330; doi:10.3389/fcell.2019.00146)
Supplement: Supplementary file 1 [file Data_Sheet_1.doc]

**Supplementary Materials**

Here, we are trying to re-interpret the corner-stone papers that are in favour of the different models of ER-to-Golgi (EGT) and intra-Golgi (IGT) transport (see also Mironov and Beznoussenko, 2019; in press).

According to the most precise electron cryo-microscopy (Bacia et al., 2011), the diameter of COPII vesicles is 65 nm to 80 nm, which is more than in other studies. Bacia et al. (2011) used electron cryo-microscopy and pure liposomes. Of interest, for the illustration of the validity of *in-vitro* data, Bacia et al. (2011) used the beaded tubule, which protrudes from the ER and does not have the typical COPII-like coat (see Fig. 3I of Bacia et al., 2011). In Lee et al. (2005), the diameter of COPII vesicles should also be 65 nm to 80 nm. Bannykh et al. (1996) also indicated that the diameter of COPII-coated buds from which COPI vesicles should be generated is >60 nm. In plants, the diameter of presumably COPII-coated buds on the granular ER varies from 60 nm to 100 nm (see Fig. 5C, D of Ritzenthaler et al., 2002, and Figs. 2A, 3A of Donohoe et al., 2007). Importantly, COPI vesicles in *S. cerevisiae* have a diameter of 50 nm (Beznoussenko et al., 2016). We would like to stress, that the vesicles accumulated by Kaiser and Schekman (1990) also had a diameter of 50 nm. Moreover, within ERES, only 50-nm completely spherical vesicles were found using a quick-freezing technique, with no 65 nm to 80 nm vesicles (Mironov et al., 2003). Also, Zeuschner et al. (2006) reported only free 50-nm-diameter vesicles. COPII-coated structures of ERES were not completely spherical. Finally, Bannykh et al. (1996) also did not find separate 65 nm to 80-nm vesicles within ERES. Additionally, we found depletion of anterograde cargoes in 50-nm vesicles localised within ERES (Mironov et al., 2003). In the *Microsporidia* parasite, the ER and GC remnants also have no such buds and separate vesicles (Beznoussenko et al., 2007).

Kaiser and Schekman (1990) generated temperature-sensitive mutants of the proteins that are responsible for EGT (COPII subunits Sec23 and Sec13) and the proteins involved into membrane fusion (Sec 17 [known also as αSNAP] and Sec18 [known also as NSF]). When the cells were warmed to 37 °C and membrane fusion was blocked (Sec17 or Sec18 did not function), they showed accumulation of 50-nm vesicles. When they simultaneously inhibited both Sec17 and Sec23, a significant accumulation of 50-nm vesicles was not observed (Fig. 3 of Kaiser and Schekman, 1990). Further, when the fusion of COPII vesicles presumably generated by Sec23 or Sec13 is blocked, COPII vesicles should accumulate, whereas when not only fusion of the vesicles with the distal compartments but also their generation is inhibited, accumulation of vesicles should not be observed; they found these phenomena.

According to the KARM, Sec23 and Sec13 are important for EGT, although COPII vesicles are very rare and do not function as ER-to-Golgi carriers. According to the KARM, when they inhibited Sec17, there should be accumulation of COPI vesicles, because COPII vesicles have no transport role. Indeed, they observed accumulation of 50-nm vesicles. Electron microscopy tomography revealed that in *S. cerevisiae* the diameter of COPI-dependent vesicles is 50 nm (Beznoussenko et al., 2016). Also, when both Sec17 and Sec23 were inhibited, there should presumably be an accumulation of COPI vesicles, although only if the Golgi complex (GC) remains separate from the endoplasmic reticulum (ER). However, in *S. cerevisiae*, when the delivery of cargo to the GC is blocked, the GC disappears in 4 min (Morin-Ganet et al., 2000), and the generation of COPI vesicles stops. In the absence of cargo delivery, the brefeldin A-induced redistribution of the GC into the ER requires membrane fusion between the GC and the post-Golgi compartments (Fukunaga et al., 1998; Kweon et al., 2004), whereas in *S. cerevisiae*, the GC can be connected with the ER (Beznoussenko et al., 2016, and reference therein), and is not dependent on membrane fusion. Indeed, in agreement with the prediction derived from the KARM, Kaiser and Schekman (1990) reported little accumulation of vesicles (from the normal 13 vesicles/µm^3^ up to 20 vesicles/μm^3^). In *S. cerevisiae*, the speed of COPI vesicle generation should be lower than in mammals, because the numeric density of COPI-dependent buds on their rims is much lower than in mammals (compare Ladinsky et al., 1999, and Beznoussenko et al., 2016). Therefore, when the function of Sec17 was blocked and the putative COPII vesicles were not formed, COPI-dependent vesicles could instead be generated from the GC.

The study of Barlowe et al. (1994) presented electron microscopy images of round profiles partially coated with a COPI-like coat (Fig. 8E of Barlowe et al., 1994) and images of cryo-sections of round profiles with diameters of 66-73 nm and labelled for Sec23 (Fig. 8G of Barlowe et al., 1994) and Sec13 (see Fig. 8H of Barlowe et al., 1994), but not for Sar1 (see Fig. 8E of Barlowe et al., 1994). In Figure 8E, the diameter of these vesicles partially coated with a COPI-like coat was very uniform, and varied from 38 nm to 55 nm, with a mean of 51 nm. This characteristic is typical of COPI vesicles (Marsh et al., 2001). Also, the structure of the vesicle coats in Figure 8E was similar to that observed for COPI-coated buds localised within ERES (Bannykh et al., 1996) and differed from the structure of the COPII coat demonstrated with electron cryomicroscopy (see Bacia et al., 2011). The vesicles shown in Figure 8B-D and 8F-H of Barlowe et al. (1994) showed much higher heterogeneity than the 51-nm vesicles in Figure 8E. These parameters are typical for membranes with a COPII coat (Lee et al., 2005). Importantly, the same research group used electron cryomicroscopy and pure liposomes to demonstrate that the size of COPII-dependent vesicles should be about 75 nm to 80 nm (Fig. 3A of Lee et al., 2005; Bacia et al., 2011). We believe that these authors demonstrated two types of vesicles: COPI-dependent (see their Figs. 7, 8E) and COPII-dependent (see their Fig. 8B-H). When the primarily isolated COPII-coated tubules were passed through small pores, they can undergo fragmentation into 66-nm COPII-coated vesicles. These vesicles do not contain Sar1, because COPII-coated buds and tubules are devoid of Sar1p (Mironov et al., 2003).

However, our hypothesis that together with COPII vesicles, COPI vesicles are also generated should be explained. For instance, COPI-dependent vesicles need COPI for their generation. Careful analysis of the experimental procedures used by Barlowe et al. (1994) revealed that they showed that their microsomes contain residual amounts of COPI attached to the membranes of the microsomes. However, in their experiments on the isolation of vesicles, they do not use the microsomes washed with 2.5 M urea to eliminate all peripheral membrane proteins. Thus, this residual amount of COPI present on their microsomes might have been sufficient to generate a few COPI-coated vesicles. If these 51-nm vesicles were formed with COPI, why are these vesicles coated? The reason is the following. The ArfGAP protein, which is necessary for COPI uncoating, is present on the GC, but not on the ER (microsomes).

Then, it is necessary to explain why transmission electron microscopy sectioning gave images of COPI vesicles, whereas cryosectioning resulted in COPII vesicles. The reason might be the following. After centrifugation of the membranes passed through small pores, they obtained a pellet where the heavy COPI-coated vesicles would be near the bottom, whereas the lighter COPII-coated structures would be above. Such a distribution was described in our study (Kweon et al., 2004). Then they embedded the pellet in Epon or prepared it for cryosectioning. The sectioning of an Epon-embedded pellet starts from the very bottom, and therefore for the sections mostly 48-50-nm partially COPI-coated round profiles would be visible. In contrast, for cryosectioning it is necessary to prepare a pyramid. Therefore, several layers of a pellet, which were situated near the bottom and contain mostly COPI, were eliminated to get the precise pyramid. As a result, for the cryosections there would only be vesicles derived from COPII-coated tubules.

Finally, in the study of Bednarek et al. (1995), in their Material and Methods, they cited Rexach and Schekman (1992) for the description of the isolation of nuclei. However, Rexach and Schekman (1992) did not use 2.5 M urea to wash the membranes of COPI. Thus, a significant amount of COPI was present on the external nuclear membrane of the ER, which can be used for the generation of COPI-coated buds on the ER.

Rizzo et al. (2013) demonstrated that after the polymerisation of their chimeric mannosidase I (ManI-FM), this protein shifted to the *trans-*side of the GC. Although the monomeric form of this chimera is depleted in near-Golgi round profiles, after the depolymerisation, this protein quickly appeared in round profiles. According to the authors, 50% of these round profiles were coated with COPI. These data were interpreted in favour of the CMPM. However, Rizzo et al. (2013) did not use serial cryosections to distinguish between the round profiles as projections of cross-sections of the tubules and projections of actual vesicles. On random cryosections, this distinction is not possible (Kweon et al., 2004). Moreover, on cryosections, a tubular network can appear as round profiles, which they considered as COPI-dependent vesicles. Also Rizzo et al. (2013) did not perform the obvious control experiment based on the inhibition of the formation of COPI vesicles (i.e., microinjection of cells with an antibody against ßCOP). Also, their tomography data presented in favour of augmentation of the number of COPI-dependent vesicles after depolymerisation are not convincing. Further, Rizzo et al. (2013) stated, “All reconstructions indicated that the morphology of the carriers (Supplementary Fig. S5h–m) and the structure and size of the Golgi stack were similar under all experimental conditions (Supplementary Fig. S5d, e). Moreover, tomography confirmed that most round, 50- to 80-nm structures were indeed vesicles (Supplementary Fig. S5h–m) and that the relative frequency of vesicles and tubules was similar to that seen in thin sections (not depicted).” To demonstrate that round profiles represent separate vesicles, they showed very small sized serial electron microscopy tomography images of only one round profile. However, this round profile has a visible neck connecting it with a Golgi cisterna. This neck is visible in frames 45-55 and in Figure S5j, k of Rizzo et al. (2013). If we take into consideration that the thickness of their tomography slice is 3 nm, and the resolution of the presented images is 10 nm, the obvious conclusion is that this neck represents a membranous structure, and that this round profile actually represents a COPI-coated bud. The statement that 50% of round profiles were coated with COPI is also in favour of our explanation, because the vast majority of free vesicles near the GC are not coated (Marsh et al., 2001). No electron microscopy tomography images showing the effects of AP12998 re-addition were presented.

Of interest, in Figure 5f, labelled round profiles are shown. However, there is no augmentation of the numbers of round profiles. On the other hand, in contrast to Figure 5b, Figure 5f demonstrates that the numbers of round profiles near the Golgi are normal. To find an explanation for these discrepancies, we also carried out some calculations. The surface area of Golgi vesicles is 16% of the total surface area of the Golgi cisternae (Ladinsky et al., 1999). The labelling density (LD) of Mani-FM over the cisternae before the addition of the AP12998 depolymeriser was 1.7 and over round profiles it was 0.38 (Fig. 5d of Rizzo et al., 2013). At 1.5 min after the addition of the AP12998 depolymeriser, the perimeter of the round profiles increased 3-fold (Fig. 5e; they counted the perimeter of the vesicles, indeed, the round profiles; however, the diameter of COPI vesicles is extremely stable at 52 nm (Marsh et al., 2001); the LD over cisternae decreased to 0.4; the LD over the round profiles increased 2.7-fold, to become 1.03. Simple calculations suggest that in the representative images, the number of gold particles over round profiles should be 2-fold higher than over cisternae. However, in Figure 5B, the number of gold particles over the round profiles is one fifth than those over the cisternae. Thus, the error here is 10-fold. Even if we decreased our assumptions in favour of the authors, the discrepancy remains obvious.

We think that it is possible to provide alternative interpretations of these data. Indeed, it has been established that ManI is localised within the highly perforated *cis-*most cisterna (Marra et al., 2007). Indeed, ManI-FP is localised mostly within the perforated zones of the *cis-*most cisterna and the first medial cisterna (Kweon et al., 2004). There, pores are surrounded by thin tubules with branches. This suggests that ManI is preferentially localised within the highly curved membranes. The sharp appearance of many monomers of ManI-FM can transform the solid Golgi cisterna, which resides near the *trans-*side of the GC, into the tubular network, which would protrude out of the Golgi stack. When the ManI-FM-containing chimera was stimulated to polymerise, this ability to reside within the perforated *cis-*most cisterna was lost, and ManI-FM oligomers were shifted to more solid cisternae at the *trans-*side of the GC. When the ManI polymers are depolymerised, the monomers of the ManI chimera cannot reside in the solid Golgi cisterna, and need cisternae with tubules, such as the *cis-*most cisterna.

**Clathrin-dependent recycling**

Finally, it is important to evaluate the proposal that clathrin-dependent vesicle could execute recycling of resident proteins from the trans most-cisterna and the TGN. Indeed, sialyltransferases and fucosyltransferases are present within the *trans-*most cisterna (TMC). However, there are no buds coated with COPI on the TMC (Ladinsky et al., 1999; Marsh et al., 2001; Mironov et al., 2017). In principle, it is possible that clathrin-dependent vesicles can function as retrograde carriers. However, this proposal has not been substantiated. Indeed, Velasco et al. (1993) observed labelling of mannosidase II with DAB in clathrin-coated buds within the GC. However, DAB labelling is not quantitative, and the conclusion whether galactosyltransferase is concentrated or depleted in this vesicle is not possible. Moreover, Rothman et al. (1980) reported that the clathrin-dependent vesicles isolated from the GC contained not Golgi-resident proteins, but the cargoes. Grabenbauer et al. (2005) did not find such vesicles with a diameter of 67 nm (the typical diameter of clathrin-dependent vesicles, which are formed on the *trans-*most cisterna and the *trans-*Golgi network (TGN). Ladinsky et al. (1999) showed that within the Golgi area there were only eight clathrin-coated vesicles. Most of the 190 uncoated vesicles had a diameter of 52 nm (see their movies). This is the diameter of COPI-dependent vesicles. It is important to underline that the diameter of COPI-dependent vesicles is very uniform (Marsh et al., 2001). If we take into consideration that the surface area of these Golgi clathrin-dependent vesicles is only 1.3-fold higher than that of COPI-dependent vesicles, the obvious suggestion is that the number of clathrin-dependent vesicle is about one twentieth of the number necessary for synchronous recycling of the Golgi-resident proteins.

Moreover, although there have been several statements that COPI-coated buds can be found within the TMC, or even the TGN, in reality this has not been completely established. There was no convincing evidence in the images that Martinez-Menarguez et al. (1999) provided that these structures were really TMC/TGN. For instance, in Figure 5B of Martinez-Menarguez et al. (1999), the coated bud labelled for ßCOP is localised at the *trans*-side of the Golgi. However, there is no clear membrane continuity of this bud with the TMC or with structures of the TGN. Thus, this image might be the section of a COPI-coated bud of the medial cisterna, and this might be an effect of the section plane. Its diameter is 60 nm. This diameter is too high for COPI vesicles. Figure 5C of Martinez-Menarguez et al. (1999) shows a membrane bud with a protein coat on the immature secretory granule within the GC of an acinar epithelial cell from the rat pancreas. However, this granule is immature and might represent a distension of the last medial cisterna. Examples of such distensions of the medial Golgi cisternae with no completely dense content can be easily found in images presented on the website “nanotomy”. Moreover, we also detected COPI-coated buds on the cargo domains, which appeared on the distension of the TMC, but only during cargo synchronisation according to the maxi-wave protocol, when a large amount of cargo moves simultaneously through the GC. Thus, everything should be considered in terms of probability, and the probability of finding COPI-coated buds on the TMC is relatively low. As such, it is not clear how the resident proteins undergo recycling from the TMC. The variability of the diameters of the COPI vesicles described by Martinez-Menarguez et al. (1996) might be a result of chemical fixation or of the methods of measurement. At least in our hands, when we inhibited SNAREs and most of the vesicles formed within the Golgi area derived from the activity of the COPI machine, their diameters were extremely uniform (Kweon et al., 2004; Fusella et al., 2013).

The fusion leads to the diffusion of lipid molecules from the distal compartment towards the thin tubules. This could induce an interaction between two different patterns of lipid order, and leads to destabilisation of these membrane tubules. These gradients between lipid domains can only occur when the interactions between the membrane components outweigh the entropic cost of sorting the lipids into domains (Safouane et al., 2010). The meeting of two lipid bilayers with different characteristics induces the rupture of the thin tubules. The increasing of membrane tension blocks the formation of tubules and destabilises those that already exist (Safouane et al., 2010). For instance, after the delivery of lipids with different physical properties, thin tubules are transformed into a row of ‘droplets’ (Mironov, 1980; Beznoussenko et al., 2015). As an example, when a water droplet falls on a water surface, it induces the formation of a thin water ‘tubule’, going upwards. This tubule is then transformed into small droplets. It might be that some of the proteins involved in membrane fission can participate in this process.

Also Omari et al. (2018) demonstrated that although most procollagen molecules that successfully move through the secretory pathway in their cells, a subpopulation did not. This subpopulation appeared in numerous dispersed puncta that co-localised with COPII subunits and autophagy markers. The numbers of LC3-positive and PCI-positive spots were smaller than the numbers of PCI-positive and LAMP1-positive spots (Omari et al., 2018). In Figure 3 of Omari et al. (2018), Sec23 and Sec31 labelling is inside the LC3 and PCI labelling. The diameter of PCI-positive spots is 2300 nm. In their Figure 3A with Cherry-Sec31 and Halo-LC3, the labelling for LC3 surrounds the labelling for Sec23 and Sec31. Similarly, in their Figure 3C, D, the labelling for LC3 surrounds the labelling for Sec23. Of interest, the labelling for PCI also surrounds the labelling for Sec23 (Omari et al., 2018). There is an exchange of Sec23 and Sec31 between these aggregates inside the PCI-positive spots and cytosol. In their Figure 5, the diameter of the PCI-positive spots that appeared from the ER and was not delivered to the GC is 1 µm, although the normal size of PCI-containing spots is 300 nm to 400 nm. In the movies presented by Omari et al. (2018), it can be seen that initially Sec23 forms an aggregate. Then, two or three PCI-containing carriers with diameters of 300 nm join with this aggregate, and the multilamellar organelle labelled for LC3 surrounds these aggregates. The final diameter becomes 1 µm. During this process, Sec23 aggregates remain accessible for exchange with the cytosolic pool of Sec23, which indicates that this membrane structure is not completely closed. In their Figure 7, the multilamellar organelle is seen inside the lysosome. This structure appeared as the late autophagosome. It contained the Sec23 aggregates, the multilamellar organelle labelled for LC3, and PCI carriers in a different position within its lumen. In their Figure 7A, the Sec23 aggregate is not co-localised with the PCI spots (Omari et al., 2018). In Movie S3 of Omari et al. (2018), PCI-positive spots are formed not inside the Sec23-positive spots, but nearby. In their movie S1, PCI-positive spots in carriers move towards the GM130-positive GC.

Our proposal is based on several similarities. Indeed, the diameter of procollagen-containing dots in Omari, McCaughey, and Gorur is significantly larger than usual ER-to-Golgi carriers that contain PCI (e.g., see movie 1 in Omari et al., 2018; movies in Patterson et al., 2008; see images of PCI spots in Mironov et al., 2003). Gorur et al. (2017) and Omari et al. (2018) also examined cells after overexpression of procollagen induced by the cell transfection. Indeed, because of the procollagen overexpression, the number of LC3-positive spots increased in normal cells (Fig. 1C of Omari et al., 2018). The labelling for Sec31 is present inside the contour of the carrier. There is no typical COPII-like coat on the cytosolic surface of the megabud. There is no cover composed of Sec31 or other COPII subunit.

**The kiss-and-run model and intra-Golgi transport in yeast**

Comparison of predictions derived from the KARM (Fig. 9) with the data by Casler et al. 2019) and Kurokawa et al., (2019) gave good fitness. Indeed, a careful frame-by-frame analysis of their movies presented by Casler et al. (2019) and Kurokawa et al., (2019) revealed that in many frames, the cargo domain and the domain of the resident Golgi proteins are clearly separated. In Casler et al. (2019), movie V4 (see also their Fig. 5) shows how the *cis-*Golgi compartment arrived at ERES. One frame demonstrates that the *cis-*domain and the cargo domains arrived simultaneously (Supplementary Fig. S3). Then the *cis-*compartment takes cargo, and moved from this place. Even in ERES, Sec31 and the cargo domain are separated in the 5th frame in movie 4 of Casler et al. (2019). In frame 11 in video V4, it can be seen that the cargo domain becomes larger. In frame 5, it can be seen that the *cis-*Golgi compartment has arrived with its own cargo domain (Supplementary Fig. S4, white arrow). In movie 5, the cargo domain and Golgi domain are not completely intermixed, but they do exist as separate domains.

In movie 2 of Kurokawa et al. (2019), there is a moment when two domains, namely, the cargo and the Golgi compartment, are well visible. Kurokawa et al. (2019) also stated: “cargo does not occupy the whole area of the maturing cisterna, but is rather in a restricted zone”. This is against the CMPM, because if the Golgi-resident proteins are concentrated within the restricted zone along the cisternal rims, the efficiency of COPI-vesicle-mediated recycling would be lower, and so it is necessary to have specific mechanisms for the concentrating of the process of COPI-dependent membrane budding within this area. Kurokawa et al. (2019) showed that pores are present not only along the rims, but also in the central zone of the Golgi cisterna. Also, in the movies of Casler et al. (2019), it is possible to visualise the arrival of the *cis-*Golgi compartment at ERES together with the cargo domain (Supplementary Fig. S3).

Casler et al. (2019) also stated, “the transient increase raised the signal by an average of 70%” (their Fig. 6C). In these Figures, it is possible to see that the increase in the cargo signal started before the arrival of the *trans-*Golgi compartment, and the amount of cargo within the *trans-*Golgi compartment after the detachment of the cis compartment is higher. Also, according to Kurokawa et al. (2019), “the amount of cargo in the maturing cisterna appeared to show a slight increase in *trans-*Golgi during the *cis-to-trans* maturation and a transient increase and decrease during the maturation from *cis-*Golgi to the TGN”. Casler et al. (2019) showed that the initial stage of the cargo domain interaction with ERES is very transient (nine frames in their Movie 3). Also, in movie 4 of Casler et al. (2019), it is clearly demonstrated that after detachment of the cargo domain from ERES, its size and intensity become greater than before (Supplementary Fig. S3). In movie 5, Casler et al. (2019) demonstrated that the transient stage is very short. We counted the numbers of frames corresponding to the different phases, and we found that the *cis-*cargo stage takes 28 frames, the transient stage takes 9 frames, and the *trans-*cargo stage takes 30 frames. However, for their Figures presented in the text, Casler et al. (2019) selected only frames where the cargo and the Golgi domains co-localised. However, this might just be the result of an over-projection effect. Also, careful frame-by-frame examination of the movies presented in the studies by Matsuura-Tokita et al. (2006), Losev et al. (2006) and Kurokawa et al. (2019) revealed that during the so-called maturation of the Golgi compartments, the stage when the mixture (yellow) of the two original colours (red, green) exits is very short. In contrast, according to the CMPM, this process should be much longer. Thus, the KARM can easily explain the data by Casler et al. (2019) and Kurokawa et al. (2019).


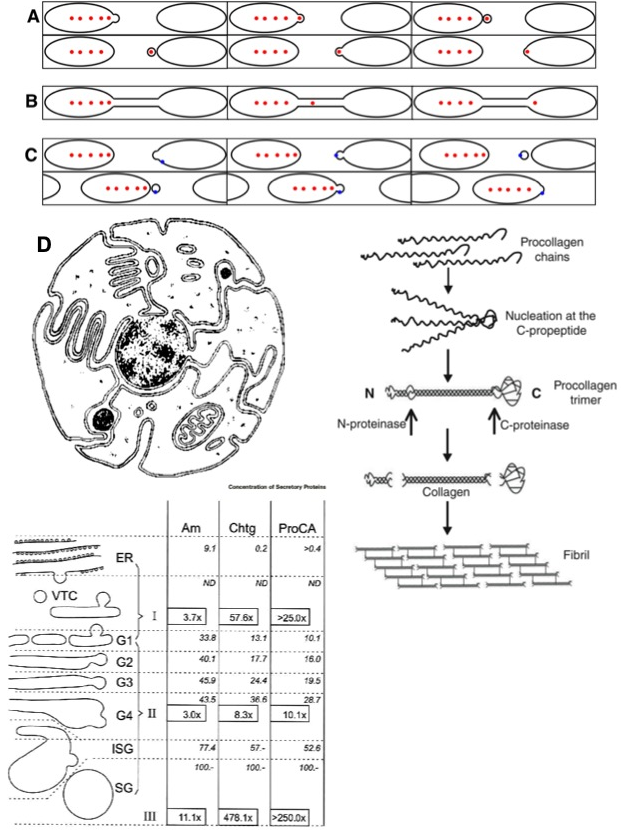


**Figure S1.** Different modes of membrane transport. **(A)** Dissipation**. (B)** Diffusion (Fig is taken crom Ro. **(C)** Progression with recycling. **(D)** The proposal by Robertson (1962) that all cellular compartments are connected. This is the basis of the DM. Right: Formation of procollagen trimers as a megacargo. Bottom: Different concentrations of cargo during intra-Golgi transport for different regulation of secretory proteins (from Oprins et al., 2001). Right: An example of the megacargo. Assembly of procollagen molecules into collagen trimers.


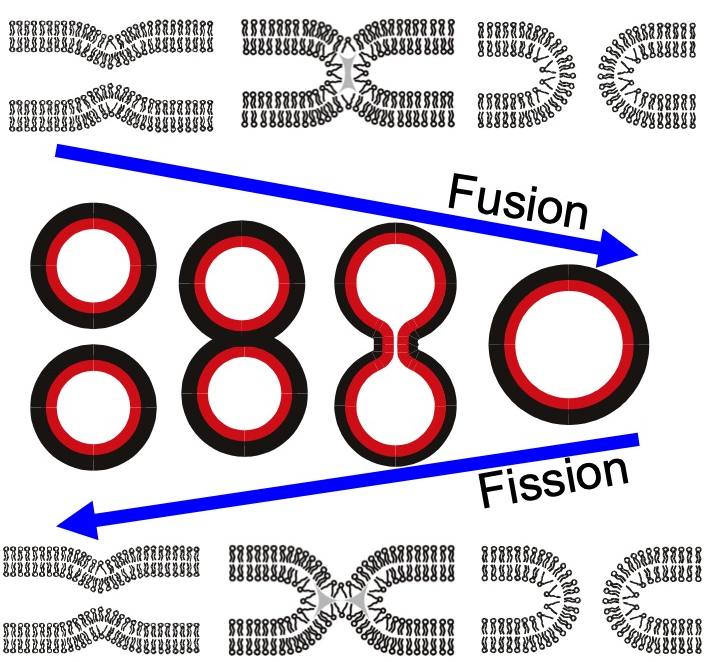


**Figure S2.** The interdependency of the fusion and fission mechanisms.


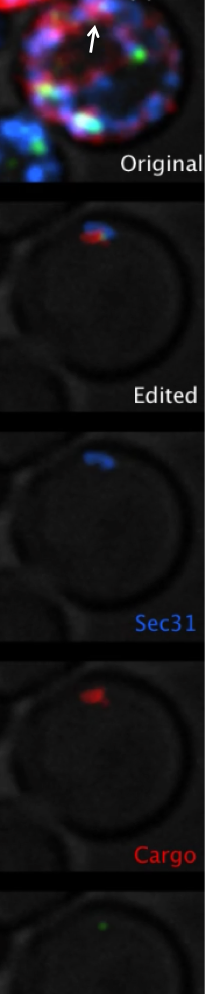


**Figure S3.** The frame from movie V4 of Casler et al. (2019) that shows that the *cis-*Golgi compartment arriving at ERES is already connected with the cargo domain (arrow).

**Additional references for the Supplementary Material**

Donohoe BS, Kang BH, Staehelin LA. Identification and characterization of COPIa- and COPIb-type vesicle classes associated with plant and algal Golgi. *Proc Natl Acad Sci U S A* (2007) **104:**163-168.

Fukunaga T, Furuno A, Hatsuzawa K, Tani K, Yamamoto A, Tagaya M. NSF is required for the brefeldin A-promoted disassembly of the Golgi apparatus. *FEBS Lett* (1998) **435:**237-240.

Losev, E., Reinke, C.A., Jellen, J., Strongin, D.E., Bevis, B.J., and Glick BS. 2006. Golgi maturation visualized in living yeast. Nature. 441(7096):1002-1006.

Mironov AA. 1980. Structural and ionometric analysis of the mechanisms of the development of irreversible injuries in the hypothermic preservation of kidneys in a solution of the intracellular type. Arkh Patol. 42(10):95.

Rexach MF, Schekman RW. Use of sec mutants to define intermediates in protein transport from endoplasmic reticulum. *Methods Enzymol* (1992) **219:**267-286.

Ritzenthaler C, Nebenführ A, Movafeghi A, Stussi-Garaud C, Behnia L, Pimpl P, Staehelin LA, Robinson DG. 2002. Reevaluation of the effects of brefeldin A on plant cells using tobacco Bright Yellow 2 cells expressing Golgi-targeted green fluorescent protein and COPI antisera. Plant Cell. 14(1):237-261.

[Safouane M](https://www.ncbi.nlm.nih.gov/pubmed/?term=Safouane M%5BAuthor%5D&cauthor=true&cauthor_uid=20887377), [Berland L](https://www.ncbi.nlm.nih.gov/pubmed/?term=Berland L%5BAuthor%5D&cauthor=true&cauthor_uid=20887377), [Callan-Jones A](https://www.ncbi.nlm.nih.gov/pubmed/?term=Callan-Jones A%5BAuthor%5D&cauthor=true&cauthor_uid=20887377), [Sorre B](https://www.ncbi.nlm.nih.gov/pubmed/?term=Sorre B%5BAuthor%5D&cauthor=true&cauthor_uid=20887377), [Römer W](https://www.ncbi.nlm.nih.gov/pubmed/?term=Römer W%5BAuthor%5D&cauthor=true&cauthor_uid=20887377), [Johannes L](https://www.ncbi.nlm.nih.gov/pubmed/?term=Johannes L%5BAuthor%5D&cauthor=true&cauthor_uid=20887377), [Toombes GE](https://www.ncbi.nlm.nih.gov/pubmed/?term=Toombes GE%5BAuthor%5D&cauthor=true&cauthor_uid=20887377), [Bassereau P](https://www.ncbi.nlm.nih.gov/pubmed/?term=Bassereau P%5BAuthor%5D&cauthor=true&cauthor_uid=20887377). Lipid cosorting mediated by shiga toxin induced tubulation. *Traffic* (2010) **11(12):**1519-1529.

Additional movies that help to demonstrate how the models work can be found at the following websites:

**Movie 1.** The vesicular model (https://yadi.sk/i/M1ykjxKabmjs5 ). For explanation, see the legend to Figure 5.

**Movie 2.** The cisternal maturation–progression model (https://yadi.sk/i/gxcOI8fvbmLz2 ). For explanation, see the legend to Figure 7.

**Movie 3.** The concentrating of quasi-soluble cargo according to the kiss-and-run model (https://yadi.sk/i/5Zb4AmGqbmjxf ). Two compartments are connected with a thin tubule. A cargo (black dots) can diffuse through the tubule. In the distal compartment, protons (red dots) are pumped into the lumen. The protons interact with the cargo and induce its oligomerisation. Oligomers cannot diffuse backwards through the tubule.
