# Supplementary figures and images for: Models of Intracellular Transport: Pros and Cons
Source: Front Cell Dev Biol. 2019 Aug 7;7:146. doi: 10.3389/fcell.2019.00146 (PMC6693330; doi:10.3389/fcell.2019.00146)

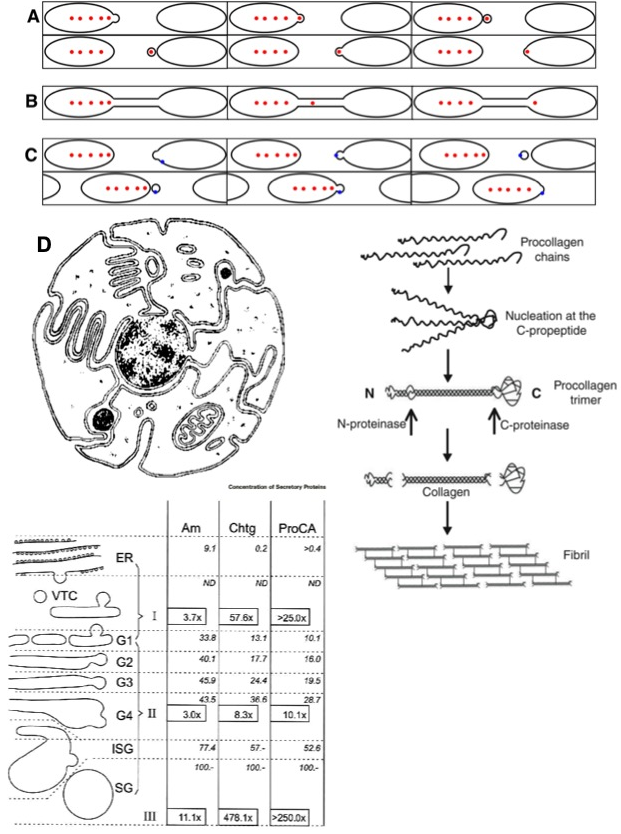

Supplement: Supplementary file 2 [file Image_1.TIFF]

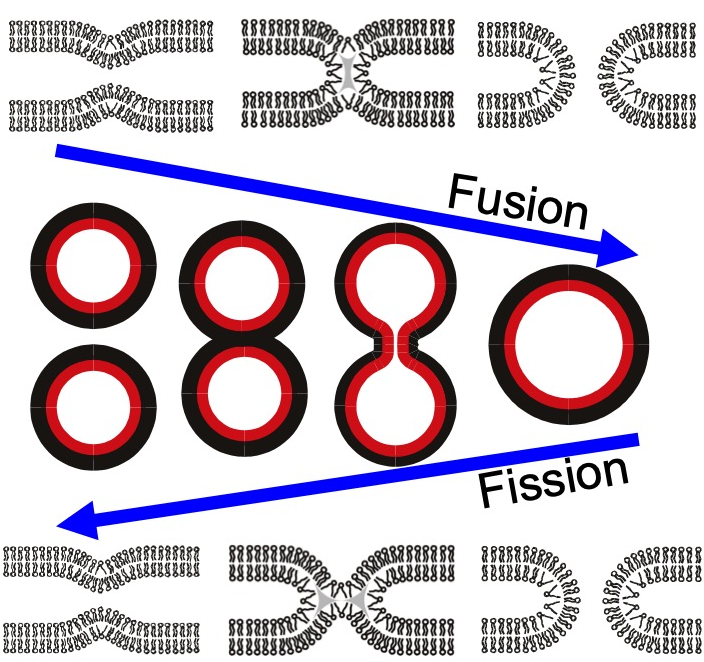

Supplement: Supplementary file 3 [file Image_2.TIF]

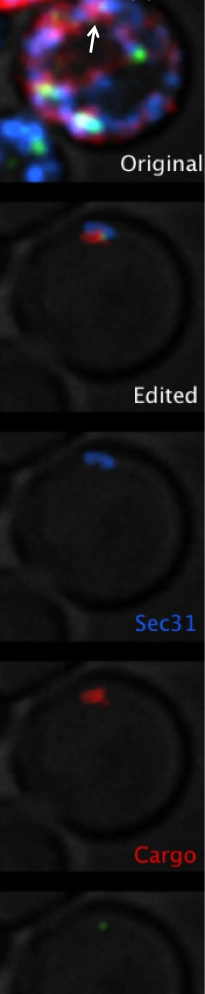

Supplement: Supplementary file 4 [file Image_3.TIFF]
